# Infectious bacterial keratitis management in a Swedish region: Risk factors, treatment and time‐to‐resolution

**DOI:** 10.1111/aos.70141

**Published:** 2026-04-12

**Authors:** Jenny Roth, Antonio Filipe Macedo, Sofia Somajo, Lukas Tannerfeldt, Baris Toprak, Neil Lagali

**Affiliations:** ^1^ Department of Medicine and Optometry Linnaeus University Kalmar Sweden; ^2^ Department and Centre of Physics‐Optometry and Vision Science University of Minho Braga Portugal; ^3^ Department of Chemistry and Biomedical Sciences Linnaeus University Kalmar Sweden; ^4^ Clinical Microbiology Region Kronoberg Växjö/Karlskrona Sweden; ^5^ Department of Biomedical and Clinical Sciences, Faculty of Medicine Linköping University Linköping Sweden; ^6^ Department of Ophthalmology Sørlandet Hospital Arendal Norway

**Keywords:** aetiology, bacteria, clinical outcomes, contact lenses, keratitis

## Abstract

**Purpose:**

To report treatment patterns, time‐to‐resolution (TTR) and aetiologies of bacterial keratitis (BK) infections treated in a specialized hospital‐based cornea clinic in Sweden.

**Methods:**

Retrospective study based on electronic health records screened during the period 2010–2019. The study included only culture‐confirmed cases of BK. TTR was defined as the number of days from the sample collection for microbiological culture to the date that the cornea healed. Factors affecting TTR were investigated with Cox regression models.

**Results:**

Contact lens wear and severely ill/blind eye were the most common aetiologies among the 188 patients. Empirical treatment consisted of fluoroquinolones in combination with aminoglycosides in 50% of the patients. Median TTR was 22 days (95% CI: 12, 34) for contact lens–related BK and 90 days (95% CI: 55, 146) in complicated cases with miscellaneous or unknown aetiology. Hazard ratio (HR) for infections in females (HR = 1.93; 95% CI: 1.34, 2.79), contact lens wearers (HR = 2.72; 95% CI: 1.79, 4.14) and infections caused by Gram‐positive pathogens (HR = 2.50; 95% CI: 1.50, 4.19) indicated that infections in these categories healed at a faster rate than their reference categories. Patients switching to treatment with chloramphenicol after it was absent in the empirical treatment had a slower rate of healing than those who received chloramphenicol as part of the empirical treatment (HR = 0.58; 95% CI: 0.38, 0.87).

**Conclusions:**

The current study provides unique TTR estimates for BK. The results revealed that age, sex, aetiology, empirical treatment and Gram staining can affect TTR. In our sample, empirical treatment that includes chloramphenicol could potentially reduce TTR. These findings can be used to individualize disease management and prognosis.

## 1 INTRODUCTION

Infectious keratitis is a serious ocular condition that can lead to corneal damage secondary to epithelial defects and corneal ulceration resulting in severe vision impairment (Puig et al., 2020; Ung & Chodosh, 2023). In North America and Europe, bacterial keratitis (BK) is the most prevalent subtype of infectious keratitis (Ting, Ho, et al., 2021; Ung et al., 2019). Contact lens wear has been consistently linked to an increased risk of contracting BK (Bourcier et al., 2003; Puig et al., 2020; Sagerfors et al., 2020). Other contributing factors include ocular surface disorders and ocular surgery such as penetrating keratoplasty (Cabrera‐Aguas et al., 2022; Khoo et al., 2020; Puig et al., 2020; Ting, Ho, et al., 2021).

Bacterial keratitis can be caused by a wide range of Gram‐positive and Gram‐negative bacteria. The predominant organisms often vary depending on geographic location and aetiology, influencing severity of infection and treatment strategies (Bourcier et al., 2003; Brown et al., 2021; Ung et al., 2019). In our recent study conducted in Sweden, we found that Gram‐positive bacteria were more common than Gram‐negative in lab‐confirmed infectious bacterial keratitis (Roth et al., 2024). A subsequent study in another region of Sweden reported similar findings (Österhed et al., 2025).

The type of bacteria and the underlying cause of BK (aetiology) may influence the efficacy of antibiotics used to treat it, with poor targeted treatment leading to prolonged time to resolution (TTR) of the infection. The longer a BK is active, the higher the likelihood that it causes irreversible corneal damage and vision impairment (Singh et al., 2022; Soleimani et al., 2021; Zhang et al., 2022). It is currently common to use surgical procedures such as collagen crosslinking or keratoplasty to repair severely damaged corneas or to promote healing in infections unresponsive to medical treatment with antibiotics (Singh et al., 2022). A successful clinical outcome of a BK treatment is the resolution of the infection, with corneal healing and preservation of good vision.

Treatment outcomes of BK can be influenced by several factors including: patient‐specific factors such as age, sex or general health (Green et al., 2007; Onyia et al., 2026); environmental factors such as types of bacteria (Green et al., 2007) and antibiotic efficacy (Thomas et al., 2019). In addition, outcomes can depend on local clinical capabilities such as access to best surgical techniques for corneal healing and repair (Ahmed et al., 2025). Given this, updated epidemiological surveillance on causes and real‐world (outside the context of clinical trials) treatment outcomes of BK is essential to identify knowledge gaps, establish research priorities, reduce the incidence and severity of vision loss and corneal damage, and guide clinical practice.

The aim of this study was to determine TTR of severe cases of bacterial keratitis and factors influencing it in patients treated at a specialized hospital‐based cornea clinic in Sweden. The study focuses on severe cases of BK defined as those requiring microbiological investigation, as these infections are expected to be more challenging to treat and pose greater risk to patients' vision. In addition, we report changes in visual acuity and complications associated with severe BK infections, including cases that required corneal surgery. By characterizing the history of these infections, this study contributes new and up‐to‐date information for the management of BK.

## 2 METHODS

### 2.1 Study sample

This retrospective study was approved by the Swedish Ethics Review Authority (Protocol number 2019‐05234). The electronic patient database was screened to identify all cases of keratitis treated between 2010 and 2019 at the specialized hospital‐based cornea clinic at Linköping University Hospital in Östergötland County, which provides secondary and tertiary care to a catchment area of approximately one million people. Information was exported from the records in cases with one or more of the following ICD10 diagnosis codes: H16: keratitis, H16.1: other superficial keratitis without conjunctivitis, H16.2: keratoconjunctivitis, H16.3: interstitial and deep keratitis, H16.8: other keratitis, H16.9: keratitis, unspecified, H19.2: keratitis and keratoconjunctivitis in other infectious and parasitic diseases classified elsewhere, H19.3: keratitis and keratoconjunctivitis in other diseases classified elsewhere.

The final sample used here was also used in a previous publication (Roth et al., 2024) where the selection process was described in detail; a summary is provided in Figure S1. Below we provide the details necessary to understand the current study.

*First manual selection*: Electronic health records of cases identified from the database were reviewed by a senior ophthalmologist (co‐author Baris Toprak, BT). Cases with suspected bacterial cause were included in the study and other types of keratitis were excluded. Suspected cases included all cases with a clinical diagnosis of bacterial keratitis, regardless of microbiological investigation confirmation.

*Second manual selection*: Suspected BK cases with microbiological investigation were identified and retained for analysis by co‐author BT. We defined severe cases as those described by the guidelines of the Swedish Ophthalmological Society as requiring microbiological investigations of corneal tissue samples. According to the guidelines it should be performed in cases of BK with corneal ulcers with 1 mm or more in diameter or atypical presentations of corneal ulcers, such as those involving deep corneal layers. The exact guidelines in effect during the study period have been replaced and are currently unavailable; however, the current national guidelines issued in 2023 by the National Working Group for Corneal Diseases [NAG Kornea] (Swedish Ophtalmological Society, 2023) and the expert consensus guideline published 22 years prior (2001) by Swedish National Board of Health and Welfare (Socialstyrelsen, 2001) are consistent. It is therefore expected that similar recommendations were in effect during the study (Document S1).

*Third manual selection*: All cases with a positive bacterial culture (i.e., culture‐confirmed BK) performed during the study period, were included in the final analysis. Electronic health records for these cases were inspected by two co‐authors (JR and LT) to retrieve information relevant for the current study.

### 2.2 Definitions

*Patients*: In some instances, both eyes of the same patient (bilateral infections) were included in the study. For statistical analysis, each patient was treated as the unit of analysis (not eyes).

*Surgical procedures*: a surgical intervention with the aim of, for example, relieving pain, promoting healing, having a bactericidal effect or preventing vision loss. Specimen collection or biopsies were excluded from this category. Some patients underwent up to four different types of surgical procedures. In some instances, patients had more than one surgical procedure conducted during the same surgical session; these procedures were counted into separate categories.

*First visit*: refers to the first date registered in the medical journal at which the patient sought care for BK at Linköping University Hospital eye clinic—this timepoint is different from TTR given below, and the first visit is used to inform about the functional recovery following treatment or irreparable functional loss caused by the infection.

*Time to resolution (TTR)*: defined as the number of days between the sample collection for microbiological culture and infection resolution (documented corneal healing). The cornea was considered healed when this was explicitly noted in the patient record, or otherwise by the date of the last clinical entry if no further information was available. Healing was defined as the absence of staining, no anterior chamber inflammation and no stromal or endothelial infiltration. For patients who underwent amniotic membrane transplantation, complete membrane dissolution was additionally required. In bilateral cases TTR was defined by the eye with the longer healing time. We defined the starting point of TTR as the moment at which infections were considered severe based on nationally accepted criteria, which require corneal samples for laboratory culture, reflecting an assumption of a consistent stage of disease across patients.

*Presenting visual acuity (PVA)* corresponds to visual acuity measured using the patient's habitual refractive correction (if any) or equivalent. PVA is different from best‐corrected visual acuity. Acuity was based on the following procedure: (a) when habitual and updated (self‐reported) correction was available from the patient—acuity was measured with that correction; (b) if the patient failed to bring the habitual correction or reported outdated correction—when corneal transparency allowed, the autorefractor values were used in a trial frame and acuity was measured. PVA at the first visit was compared with PVA at the end of the TTR. Acuity is reported in logMAR. Based on the WHO ICD‐11 classification we classified cases of counting fingers, hand movement, light perception and no light perception with a visual acuity of ≥1.8 logMAR (World Health Organization (WHO), 2025). For bilateral infections, PVA of the eye with the longer healing time was used.

### 2.3 Statistical analysis

A range of statistical tests were used and are reported alongside their results. For the survival analysis, the event of interest was corneal healing, defined as ‘time to resolution’ (Amorim & Cai, 2015). This was measured as the number of days between the sample collection for microbiological culture and the resolution of infection.

Kaplan–Meier curves were used to visualize the probability of an active BK infection over time. The impact of independent predictors on time to resolution (TTR) was assessed using Cox proportional hazards regression, conducted in R (coxph function, survival package) (Andersen & Gill, 1982; Therneau, 2024; UCLA: Statistical Consulting Group). For these analyses, some aetiologies were merged differently from the descriptive statistics to improve interpretability without compromising conclusions. Hazard ratios (HRs) greater than 1 with *p* < 0.05 indicate a faster resolution of infection compared to the reference category. Conversely, HRs below 1 suggest a slower resolution. Additional statistical analysis was performed with SPSS Version 29.0 (IBM Inc., Armonk, NY). A *p*‐value of less than 0.05 was considered statistically significant.

## 3 RESULTS

### 3.1 General characteristics of the sample

Laboratory results revealed that 188 patients had culture‐confirmed BK. The median age of the patients was 62 years (IQR = 39–79), and 103 (55%) were females. Of the included patients, 22 were referred by general practitioners to Linköping eye clinic, whereas 15 were referred from ophthalmology outpatient clinics in smaller towns, all for further ophthalmologic assessment.

Reporting infections by eye, 93 (47%) infections were found in the right eye and 104 (53%) in the left eye, in 1 (0.5%) patient the eye was unknown. Ten patients had bilateral BK. Consistent with local practice, 81 patients (43%) were hospitalized, primarily due to intensive antibiotic treatment in combination with limited ability or support to self‐administer eye drops, with admission decisions made on a case‐by‐case basis. 105 (56%) were outpatient only, and in 2 (1%) patients it was unknown if there was hospital admission. Fifteen (8%) patients came to Linköping University Hospital for diagnosis but returned to their original clinics, and thereafter, data on treatments and infection resolution were unavailable for the current study.

Contact lens wear was the most prevalent aetiological factor, followed by a severely ill or blind eye (typically with one or multiple comorbidities). Table 1 gives a summary of the patients divided by aetiology.

**TABLE 1:** Aetiologies of bacterial keratitis and the number of patients in each category.

Aetiologies	Frequency, *n* (%)
Contact lens wear	55 (29.0)
Severely ill or blind eye^a^	39 (21.0)
Ocular surface disorder	24 (13.0)
Ocular trauma or foreign body in the cornea	13 (7.0)
Penetrating keratoplasty	10 (5.0)
Herpes simplex keratitis	9 (5.0)
Blepharitis	7 (4.0)
Eyelid disorder^b^	6 (3.0)
Severe immune deficiency	5 (3.0)
Unknown	20 (10.0)

^a^ Patients with, for example, late‐stage or neovascular glaucoma or complications after intraocular surgery.

^b^ Patients with lagophthalmos, ectropion, entropion or similar problems.

### 3.2 Microbiological profile and treatment

Of the 188 patients, 154 (82%) cultures revealed Gram‐positive bacteria, 27 (14%) Gram‐negative, and 7 (4%) both types. In 22 (12%) patients, more than one species of bacteria was reported by the microbiology laboratory, resulting in a total of 222 bacterial findings. The three most common species were coagulase‐negative staphylococci with 88 (39%) cultures, followed by *Staphylococcus aureus* with 37 (17%) and *Cutibacterium acnes* with 21 (9%) cultures. The most reported Gram‐negative bacteria were *Pseudomonas* spp., found in 15 (7%) cultures. The full microbial profile and their antibiotic susceptibility is available from our previous report (Roth et al., 2024).

Fluoroquinolones in combination with aminoglycoside were the most common empirical treatment used in 94 (50%) patients. The second most common empirical treatment was the combination of fluoroquinolone and chloramphenicol used in 23 (12%) patients. In 5 (3%) patients, information about the exact antibiotics prescribed was unavailable. The remaining 66 (35%) patients received a wide variety of antibiotic regimens including, for example, cephalosporins, macrolides and tetracyclines. Eight patients (4%) received topical antibiotics prior to corneal scrapings. In 70 (37%) patients, the treatment changed because of antibiotic susceptibility results or unsatisfactory treatment response. At least one surgical procedure was performed in 48 (26%) patients, while 16 (9%) underwent more than one surgical session.

### 3.3 Time to resolution

The median TTR of BK was 50 days (IQR: 17–126). Infections caused by Gram‐negative species had a median TTR of 71 days (95% CI: 33, 209), while infections caused by Gram‐positive species had a median TTR of 47 days (95% CI: 37, 70). Males had a median TTR of 74 days (95% CI: 47, 124), and females had a median TTR of 39 days (95% CI: 33, 59). These results are shown in Figures 1 and 2, with statistical testing across subgroups provided in Table 2.

**FIGURE 1:**
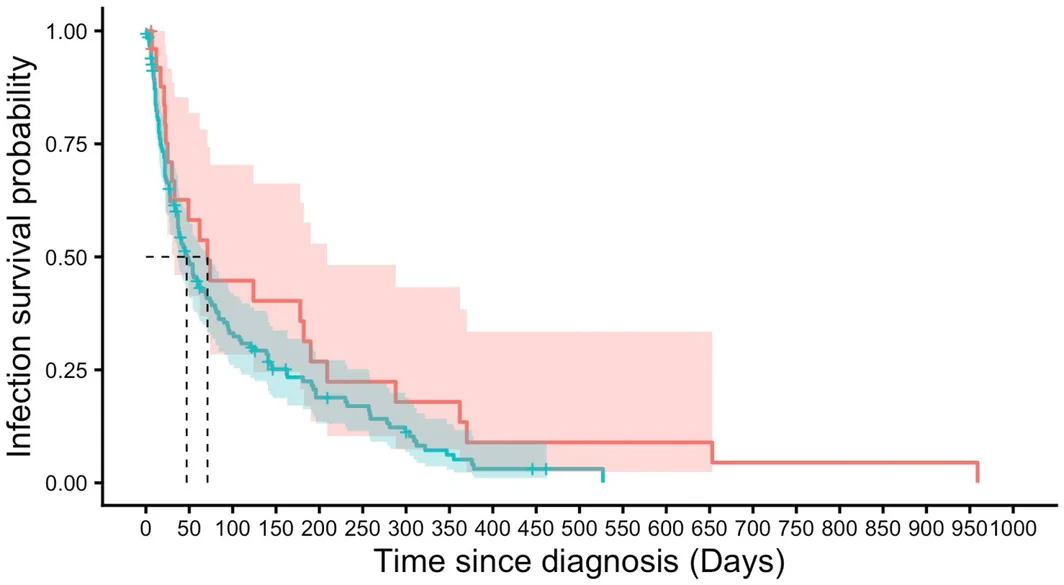
Kaplan–Meier curve showing the infection resolution probability over time for Gram‐positive (bottom curve) and Gram‐negative (top curve) bacteria. The shaded areas represent 95% confidence intervals. The dashed lines indicate the median TTR, i.e., the time by which 50% of infections had healed. Censored patients are marked with a ‘+’, indicating observations for which the event of interest (resolution of the infection) did not occur during the available follow‐up.

**FIGURE 2:**
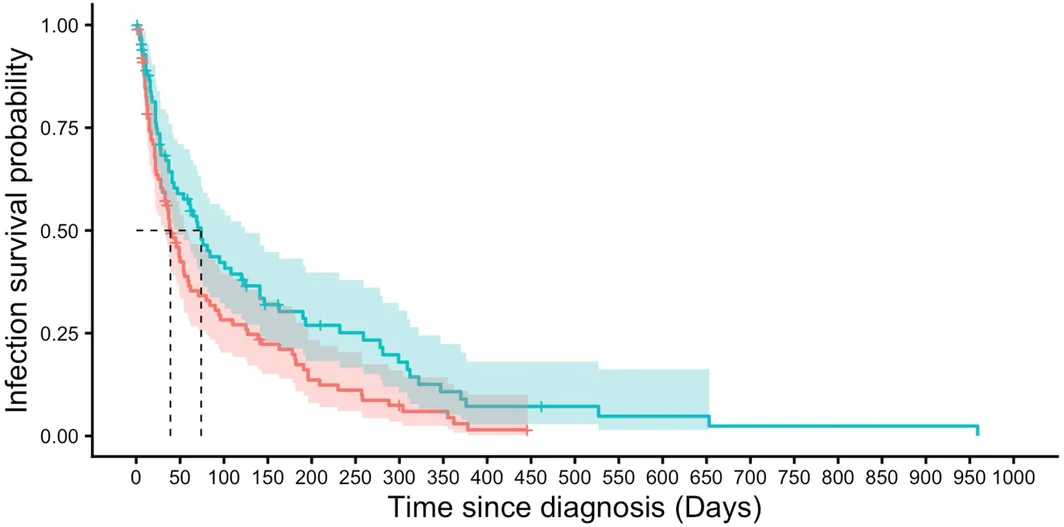
Kaplan–Meier curve showing infection resolution probability over time for females (bottom curve) and males (top curve). The shaded areas represent 95% confidence intervals. The dashed lines indicate the median TTR, i.e., the time by which 50% of infections had healed. Censored patients are marked with a ‘+’, indicating observations for which the event of interest (resolution of the infection) did not occur during the available follow‐up.

**TABLE 2:** Summary of the Cox proportional hazards regression model, with characteristics: age, sex, Gram‐stain, aetiology and antibiotic treatment as independent predictors of TTR for BK in our sample.

Characteristic	HR	95% CI	*p*‐value
Age	0.99	0.98, 0.99	**<0.001**
Sex			
M (reference)	–	–	
Female	1.93	1.34, 2.79	**<0.001**
Gram‐stain			
Gram‐negative (reference)	–	–	
Gram‐positive	2.50	1.50, 4.19	**<0.001**
Aetiology			
Miscellaneous/unknown (reference)	–	–	
SI/B	1.54	0.90, 2.64	0.120
OSD	1.44	0.83, 2.49	0.200
CLW	2.72	1.79, 4.14	**<0.001**
Antibiotic treatment			
Started with chloramphenicol (reference)	–	–	
Switched to chloramphenicol	0.58	0.38, 0.87	**0.008**
No chloramphenicol treatment	1.00	0.65, 1.54	>0.900

*Note*: ‘Started with chloramphenicol’ was used as the reference category since the guidelines recommend empirical treatment with chloramphenicol. The bold values indicate statistical significance, i.e., highlighting where the *p*‐values were less than 0.05.

Abbreviations: CI, confidence interval; HR, hazard ratio.

When comparing TTR among different aetiologies, BK associated with contact lens wear (CLW) had the shortest median TTR (22 days; 95% CI: 12, 34), compared to other aetiologies. BK with miscellaneous or unknown (Misc./unknown) aetiologies had the longest median TTR at 90 days (95% CI: 55, 146), followed by BK associated with ocular surface disease (OSD) that had a median TTR of 61 days (95% CI: 39, 232), with the wide confidence interval indicating heterogeneity within this category and variability in outcomes. BK in severely ill or blind (SI/B) patients had a TTR of 59 days (95% CI: 37, 141). These results are shown in Figure 3, with statistical comparisons between subgroups provided in Table 2.

**FIGURE 3:**
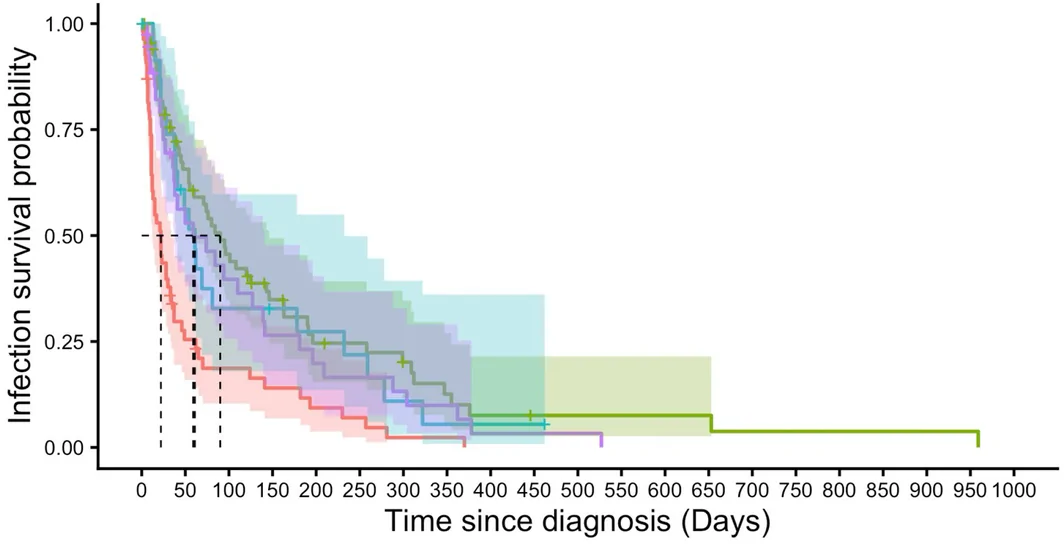
Kaplan–Meier curves showing infection resolution probability over time for bacterial keratitis with different predisposing aetiologies. The bottom curve corresponds to contact lens wear related keratitis. The top curve corresponds to miscellaneous or unknown aetiologies. The two overlapping middle curves represent ocular surface disorders and severely ill or blind eyes. The shaded areas represent 95% confidence intervals. The dashed lines indicate the median TTR, i.e., the time by which 50% of infections had healed. Censored patients are marked with a ‘+’, indicating observations for which the event of interest (resolution of the infection) did not occur during the available follow‐up.

Cox regression analysis revealed that older age significantly lengthened TTR. Each additional year of age was associated with a 1% decrease in the rate of healing (Table 2). Infections in females resolved at nearly twice the rate, and infections due to Gram‐positive pathogens at more than twice the rate, relative to the reference categories given in Table 2. Infections associated with CLW resolved at a significantly faster rate than those related to other aetiologies. Patients whose treatment was changed to include chloramphenicol after initially being treated without it had a significantly lower healing rate than those who received chloramphenicol empirically.

We performed several sensitivity analyses. In one Cox regression, we included ‘Surgical intervention’ as a characteristic. The characteristic violated the proportional hazard assumption (see Table S1), and the model is not reported. The surgical procedures sub‐sample is reported separately in the sections below. We performed a sensitivity analysis to our results by running a Cox regression model in which we excluded patients treated with an AMT (see Table S2). The results remained consistent with the model reported in Table 2. As part of the sensitivity analysis, we also ran a Cox model in which we excluded 8 patients who received topical antibiotics prior to corneal scrapings (see Table S3), and the results remained consistent with the model reported in Table 2.

### 3.4 Changes in presenting visual acuity (PVA) for the complete sample

The median PVA at the first visit was 1.0 logMAR (IQR: 0.15–1.80) and 0.4 logMAR (IQR: 0.00–1.80) at the time of corneal healing, among the 139 patients in whom PVA was measured at both time points, the improvement was statistically significant (Wilcoxon test, *Z* = −4.76, *p* < 0.001).

### 3.5 Contact lens wearers sub‐sample

Most contact lens–related infections (85%, *n* = 47) were treated with antibiotics only, but some (15%, *n* = 8) required surgical procedures. Contact lens wearers were younger than non‐contact lens wearers, and *Pseudomonas* spp. was more frequently isolated in this group. Table 3 summarizes the characteristics of contact lens wearers compared with non‐contact lens wearers.

**TABLE 3:** Characteristics and differences between contact lens–related and non‐contact lens–related BK infections based on the total 222 bacterial findings.

Characteristics	CLW (*n* = 55)	Non CLW (*n* = 133)	*p*‐value
Age (years); median	39 (IQR = 33–53)	72 (IQR = 54–84)	**<0.001**
Female sex	*n* = 33 (60%)	*n* = 70 (53%)	0.356
Pathogens	CoNS: *n* = 26 (47%) *Pseudomonas* spp.: *n* = 10 (17%) *S. aureus*: *n* = 7 (12%) *C. acnes*: *n* = 5 (9%)	CoNS: 61 (46%) *Pseudomonas* spp.: *n* = 5 (3%) *S. aureus*: *n* = 30 (18%) *C. acnes*: *n* = 16 (10%)	0.646 **<0.010** 0.123 0.561

*Note*: Differences between counts were compared with the chi‐square test (*p*‐value in the last column). The results of the chi‐square test must be interpreted with caution when counts are 5 or less. The bold values indicate statistical significance, i.e., highlighting where the *p*‐values were less than 0.05.

The median PVA for contact lens wearers at the first visit was 0.10 log MAR (IQR: 0.00–0.40) and 0.00 logMAR (IQR: 0.00–0.19) at the time of corneal healing, among 43 patients in whom PVA was measured at both time points; the improvement was statistically significant (Wilcoxon test, *Z* = −2.70, *p* = 0.007).

### 3.6 Surgical procedures sub‐sample

The most common bacterial species in the 48 patients subjected to surgical procedures were coagulase negative staphylococci (39%, *n* = 19). Patients in this sub‐sample had a median age of 77 years (IQR: 62–85), significantly older than the remaining sample with a median of 54 years (IQR: 35–78) (Mann–Whitney *U*, *Z* = −4.63, *p* < 0.001). Three‐quarters (75%, *n* = 36) of this sub‐sample had a VA of ≥1.8 logMAR at the first visit. The most common ocular comorbidity was glaucoma (30%, *n* = 14), followed by systemic hypertension (29%, *n* = 14) and diabetes (25%, *n* = 12). Table 4 summarizes the surgical procedures performed in the sub‐sample.

**TABLE 4:** Summary of the surgical procedures performed in the sub‐sample.

Surgical procedure	Performed (*n*)	Surgery sub‐sample (*n* = 48)	All cases (*n* = 188)
Amniotic membrane transplant	20	40%	11%
Crosslinking	16	33%	9%
Penetrating keratoplasty	9	19%	5%
Evisceration	6	12%	3%

## 4 DISCUSSION

The current study characterized clinical outcomes of bacterial keratitis treated in a specialized hospital‐based cornea clinic in Sweden. This study provides updated estimates of TTR from a spectrum of infections and patients that show a broader picture than the highly selective patients treated in clinical trials. The new knowledge can be used to guide current and future BK treatment strategies.

One of the most important findings of the current study is the estimates of TTR of BK, particularly the breakdown by aetiologies. Our median value of 22 days for contact lens–related infections and 90 days in complicated cases with miscellaneous or unknown aetiology is in line with previous results from clinical studies (Suresh et al., 2024). In a recent review, McDonald et al. (2014) compiled high‐quality clinical trials and reported a TTR for BK cases in the trials between 21 and 37 days, some requiring a 90 days follow‐up. Clinical trials report TTR for an average (the exact value depends on the trial) success rate of healing of about 80% (Austin et al., 2017; McDonald et al., 2014). In the present study, as illustrated in Figures 1, 2, 3, we report the median TTR, representing the time point at which 50% of cases have healed; therefore, it is important to consider the respective 95% confidence intervals. When comparing these values, it is apparent that a significant percentage of real‐world BK infections will take longer to heal than in clinical trials. This difference is likely due to a range of comorbidities, ages and causative agents that are not represented in clinical trials due to typical strict inclusion and exclusion criteria. In addition, trials tend to monitor, for example, treatment compliance which is not performed in everyday clinics. Our TTR values are relevant for clinicians to predict disease progression in routine clinical practice and to plan follow‐up assessments according to the clinical scenario of individual patients.

Our results revealed that BK was often associated with contact lens use among a younger sub‐sample of patients. Subgroup analysis revealed that treatment outcomes were good in contact lens wearers with the shortest TTR and final VA within a normal range. The association between contact lens and corneal infections has also been reported in other studies (Green et al., 2008; Khoo et al., 2020; Ung et al., 2019), and several explanations have been proposed for this association (Beshtawi et al., 2022; Ung & Chodosh, 2023; Yee et al., 2021). The explanations can be categorized into two mechanisms: increased contact between the ocular surface and pathogens, and temporary disruption of the cornea's normal surface integrity—both facilitated by contact lens use. Clear instructions on proper lens wear, handling and hygiene are essential to reduce the risk of BK among contact lens wearers.

Infections in contact lens wearers had a shorter TTR compared to other aetiologies. Consistent with these findings, previous studies have shown that individuals with contact lens–related BK tend to be younger and healthier than non‐contact lens wearers with BK (Roth et al., 2021). Younger and healthier patients are therefore more likely to experience faster healing (Gould et al., 2015). It can also be speculated that contact lens wearers' familiarity with touching the eye and surrounding areas may facilitate the self‐administration of antibiotic drops and enhance treatment compliance and efficacy.

Chloramphenicol is primarily a bacteriostatic agent often used for anterior eye infections that provides good coverage against many Gram‐positive cocci (Aramă, 2020; Bale et al., 2023). Chloramphenicol, used in combination with quinolones which are bactericidal, is recommended as empirical treatment for BK by the current guidelines (information regarding the guidelines can be found in the methods section). This combination broadens coverage and reduces the risk of undertreatment. Aminoglycosides may be used when there is strong suspicion of *Pseudomonas* infection, but because they are epithelio‐toxic, they were excluded from the empirical treatment (Swedish Ophtalmological Society, 2023). Results from the current study revealed that the combination fluoroquinolones and aminoglycoside was the most common empirical treatment even without evidence that the bacterial species involved was *Pseudomonas*. The deviation from the guidelines likely reflects reliance on clinic specific evidence and tradition for the use of fluoroquinolone–aminoglycoside combination.

In our analysis we stratified patients into three categories based on the use of chloramphenicol during the treatment of their infections. This analysis adds limited evidence, but it does provide some indication of whether the Swedish clinical guidelines for empirical treatment are still up‐to‐date. Our results revealed that patients who were switched to chloramphenicol after empirical treatment without it healed at a slower rate compared to those who received chloramphenicol from the start. The extended TTR may be attributed to aminoglycosides having a narrower spectrum of action and being less effective against Gram‐positive species than chloramphenicol, but further studies are warranted to directly compare these two empirical treatment modalities.

About 1 in 3 patients changed antibiotics after empirical treatment; this number aligns with previous studies and emphasizes the importance of laboratory testing and medical monitoring until corneal infections are resolved (Perez et al., 2012; Wenzler et al., 2023). Treatment of severe cases of BK must combine evidence‐based empirical treatment, microbiology laboratory testing and close monitoring of disease progression.

Older age was associated with a prolonged TTR and requiring surgical treatment including evisceration. These findings agree with past studies showing that elderly patients have a significantly slower healing rate (Ting, Henein, et al., 2021) and are also more likely to require surgical treatment and experience corneal perforation following infectious keratitis (van der Meulen et al., 2008). The elderly are also more likely to have comorbidities, immune system changes and ocular surface disease—alone or in combination these factors compromise defence against infections like BK (Gould et al., 2015; Kim et al., 2024). Our results revealed that systemic (e.g., diabetes) or ocular (e.g., glaucoma) comorbidities were common in patients requiring surgery. This group needs special attention for their increased risk of severe infection, poor medication adherence or both (Muñoz‐Villegas et al., 2023). In short, age and comorbidities like glaucoma are important prognostic factors for BK resolution and must be considered when defining treatment regimens.

Amniotic membrane transplantation and corneal collagen crosslinking (PACK‐CXL) were performed often. These procedures aim to promote healing and stabilize the cornea and can have anti‐inflammatory and antibacterial benefits (Austin et al., 2017; Dakhil et al., 2017; Davis et al., 2020), which can potentially reduce TTR (Ting et al., 2019; Ting, Henein, et al., 2021). In other words, surgery may support corneal healing, minimize scarring and reduce the need for prolonged use of antibiotics. PACK‐CXL has been shown to be effective even against antibiotic‐resistant strains; however, there is a need for more evidence for this treatment modality (Davis et al., 2020; de Paiva et al., 2022; Makdoumi & Bäckman, 2016; Olshaker et al., 2023).

TTR was shorter for females than for males; these findings are in line with past reports (Das et al., 2007). Reports from primary health care have shown that females generally seek medical attention earlier, more frequently, and for less severe complaints than males (Höhn et al., 2020). In ophthalmic conditions, females have also been shown to have better treatment compliance than males (Muñoz‐Villegas et al., 2023). Given the insufficient biological evidence for male/female differences in corneal healing (McKay et al., 2022), we speculate that early diagnosis and better adherence to treatment could possibly explain the faster healing times observed among females.

The current study has both strengths and limitations. A strength is that it provides a comprehensive characterization over a 10‐year period in a region, which increases knowledge about treatment practices, TTR and real‐world outcomes of treated infectious keratitis. The main weakness is the retrospective design of the study, which resulted in missing data from the patient records and reduces the precision of TTR. TTR was also influenced by follow‐up intervals determined by the treating physician; the experience of the physician, as well as the clinical course, may have influenced the TTR. Moreover, interpretation of culture results is challenging in retrospective studies; nonetheless, given that the cornea typically harbours relatively few microorganisms, any positive microbial growth consistent with known pathogens must be considered as potential causative agents, evaluated alongside the clinical presentation and clinician judgement. The division into relatively small sub‐samples was an option of the study to provide more information, but the size limits the extent of the conclusions. Future prospective studies utilizing standardized data collection methods are required to further confirm and expand upon these findings.

In conclusion, the current study provides unique estimates of TTR for BK, highlighting the heightened risk for delayed healing in the elderly, males, and in patients with Gram‐negative infections. Deviations from the empirical treatment guidelines, using aminoglycosides instead of chloramphenicol, may have an impact on TTR. Stratification of patients based on risk groups for TTR may lead to improved treatment outcomes for BK.

## ETHICS STATEMENT

The study was conducted in accordance with the Declaration of Helsinki and approved by the Swedish Ethical Review Authority (protocol number 2019‐05234).

## Supporting information


Figure S1.

Document S1.

Table S1.

Table S2.

Table S3.
