# Supplementary material for: Infectious bacterial keratitis management in a Swedish region: Risk factors, treatment and time‐to‐resolution
Source: Acta Ophthalmol. 2026 Apr 12;104(7):802–10. doi: 10.1111/aos.70141 (PMC13651938; doi:10.1111/aos.70141)
Supplement: Supplementary file 1 [file AOS-104-802-s001.pdf]

Current National Guidelines (2023)

Title: Infectious keratitis caused by bacteria, fungi and protozoa [in Swedish]

Author: National Working Group for Corneal Diseases (NAG Kornea)

Published via: Swedish Ophthalmological Society / 1177 Vårdguiden

URL: https://vardpersonal.1177.se/kunskapsstod/kliniska-kunskapsstod/infektios-keratit-orsakad-av-bakterier-svamp-och-protozoer/?selectionCode=profession_specialiserad_vard

Accessed: 22 December 2025

Infektiös keratit orsakad av bakterier, svamp eller protozoer

-State of the Art

Socialstyrelsen

Socialstyrelsen klassificerar från och med år 2001 sin utgivning i olika dokumenttyper. Detta är ett **underlag från experter**. Det innebär att det bygger på vetenskap och/eller beprövad erfarenhet som tas fram av huvudsakligen externa experter på uppdrag av Socialstyrelsen. Experternas material ger underlag till myndighetens ställningstaganden.
Författarna svarar själva för innehåll och slutsatser.

Dokumentdatum: 2002-02-28

Artikelnummer:2001-123-70

Innehåll

Definition ... 3
Infektiös keratit ... 3
Epidemiologi ... 5
Incidens ... 5
Etiologi och patogenes ... 7
Etiologi ... 7
Patogenes ... 10
Patofysiologi ... 12
Riskfaktorer allmänt ... 12
Riskfaktorer svampkeratit ... 12
Riskfaktorer Acanthamöbakeratit ... 13
Symtom och klinisk bild ... 14
Utredning/diagnostik ... 16
Odling ... 16
Mikroskopi ... 17
Behandling ... 19
Naturalförlopp ... 19
Medikamentell behandlig av bakteriella keratiter ... 19
Medikamentell behandling av svampkeratiter ... 20
Medikamentell behandling av Acanthamöbakeratit ... 21
Referenser ... 23

Definition

Infektiösa keratiter kan indelas i tre huvudgrupper: 1) Exogen keratit orsakad av bakterier, svamp eller protozoer, 2) exogen viruskeratit och 3) endogen, recidiverande viruskeratit. Dessa tre grupper har skilda kliniska bilder, patofysiologisk bakgrund, utredning och behandling. Detta dokument behandlar enbart den första gruppen, dvs exogena keratiter orsakade av bakterier, svamp eller protozoer. I den följande texten kommer denna grupp att betecknas infektiös keratit .

Infektiös keratit

Detta tillstånd är en avgränsad infektion på kornea (Fig. 1). I vårt klimatområde är infektionen vanligtvis enstaka, dvs man ser en härd och enbart ena ögat är drabbat. Spridningen sker lokalt genom epitelet (ingångsstället) till korneas stroma och eventuell till främre kammaren och/eller sklera. En keratit är inte alltid associerad med en konjunktivit (1).

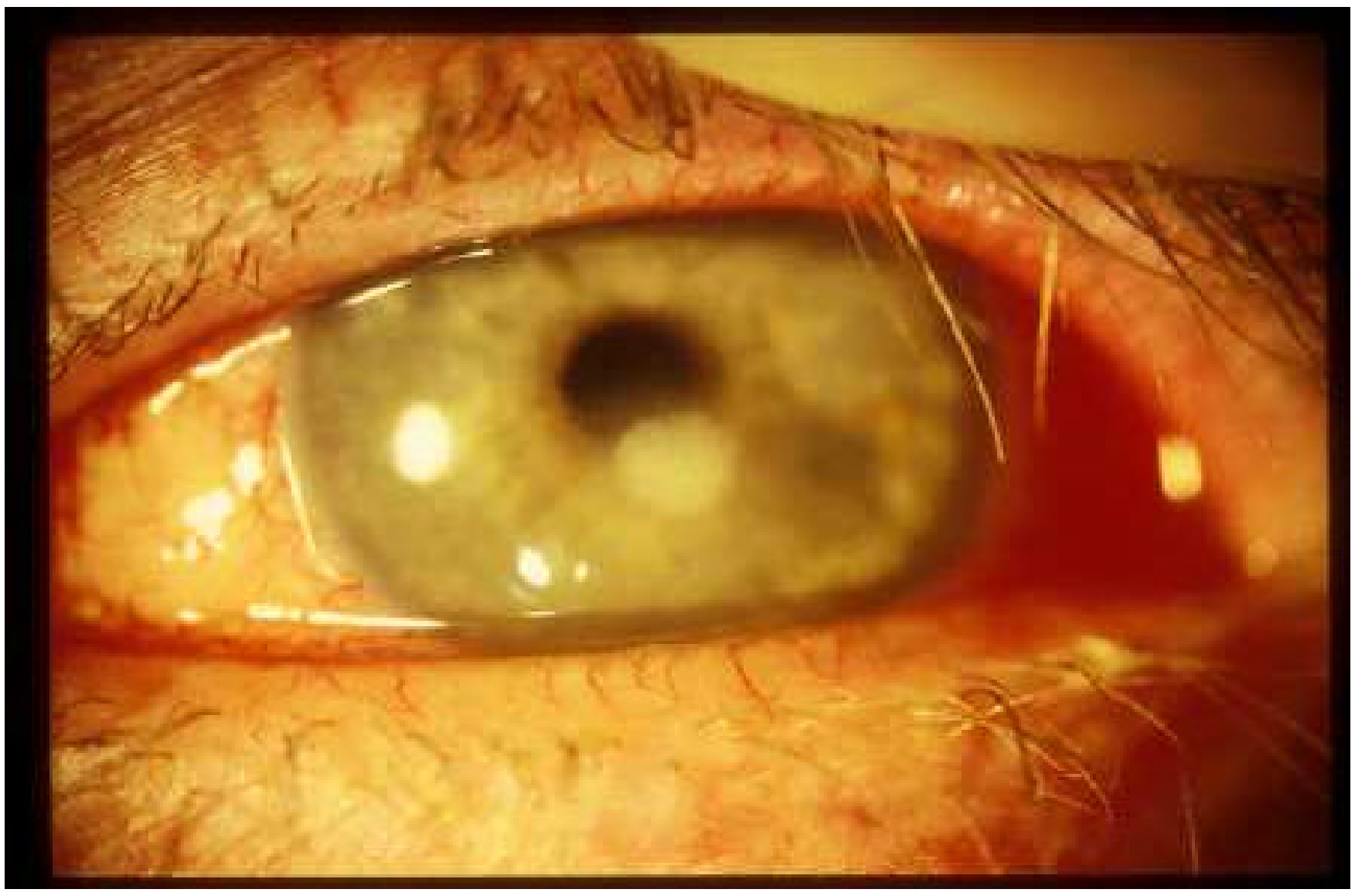

Figur 1. Avgänsad keratit

Bakterier är vanligaste etiologiskt agens i Sverige, och hittas i minst 95 % av de prov som ger positiva odlingsfynd. Svamp och protozoer är betydligt mer sällsynta och förekommer uppskattningsvis i < 2 % vardera (2, 3).

Den typiska kliniska bilden är en epitelskada i avaskulär kornea med varierande grad av underliggande ljus smältning i korneas stroma omgivet av varierande grad av ödem i kringliggande epitel och stroma. Ju djupare infektionen angripit kornea desto kraftigare inflammatorisk reaktion ses i ögats främre kammare med ansamling av inflammatoriska celler, fibrinutfällning och ibland sk hypopyon.

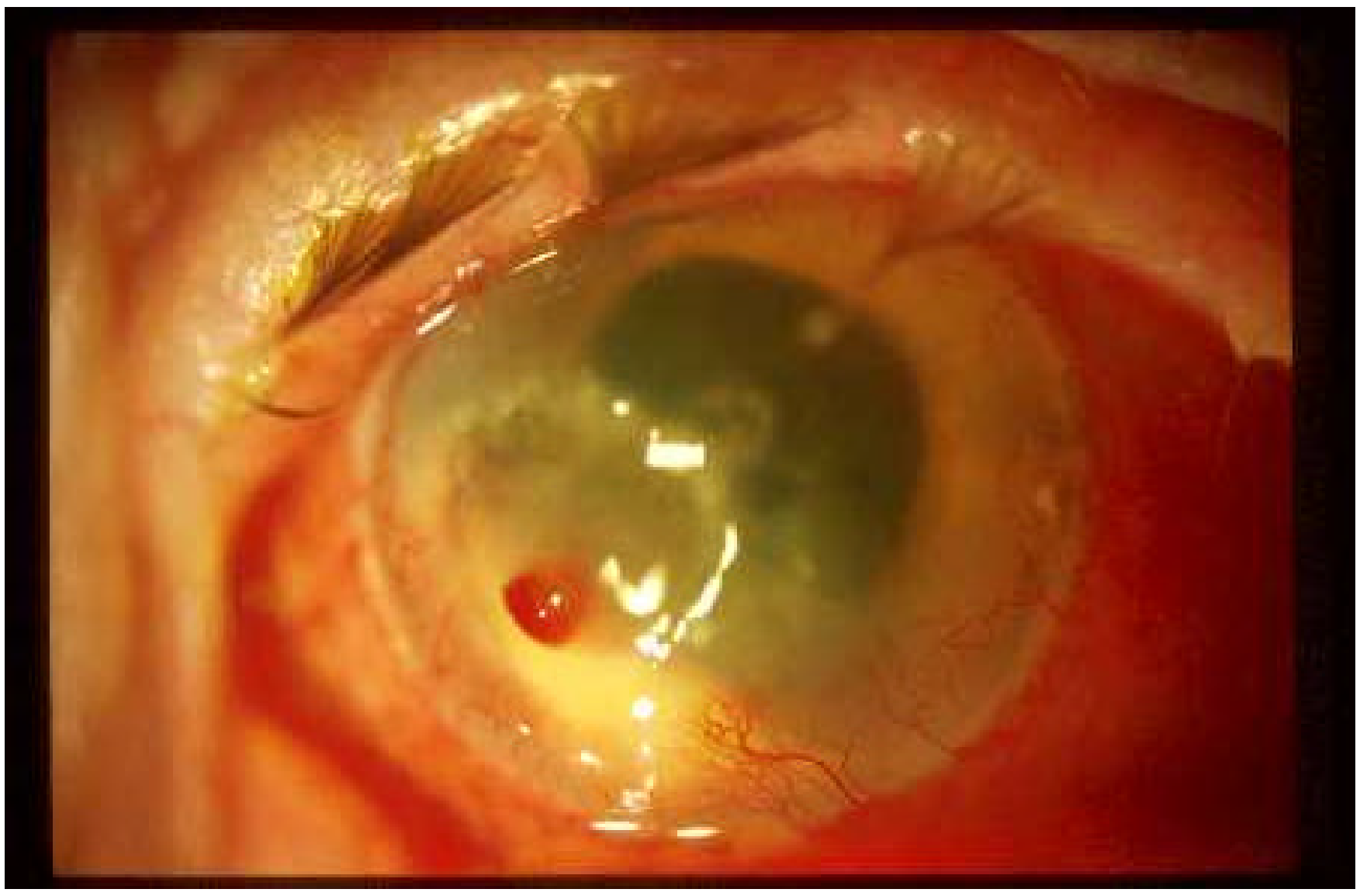

Figur 2. Djup keratit med hypopeon

Tidsförloppet kan ge en fingervisning om infektiöst agens. Snabba förlopp (timmar till enstaka dygn) talar för högvirulenta organismer såsom t.ex. *Pseudomonas aeruginosa*, medan långsamma förlopp (veckor till månader) talar för mer långsamväxande organismer såsom svamp, mycobakterier eller *Acanthamöba* (4). För att fastställa infektiöst agens rekommenderas sedan 1970-talet att man tar prov för odling och mikroskopi från keratithärden(4).

S.k. marginalkeratit kan i det enskilda fallet vara omöjlig att särskilja från infektiös keratit. Det faktum att odlingar från marginalkeratiter är sterila, att de alltid är lokaliserade limbusparallellt, att de recidiverar och oftast är associerade till kronisk stafylokockblefarit underlättar differentialdiagnostiken (5).

Epidemiologi

Risken att drabbas av infektiös keratit är beroende av faktorer som klimat, individens nutritionsläge och socioekonomiska situation, annan predisponerande ögonpatologi och kontaktlinsbärande.

I utvecklingsländerna är infektiös keratit ffa de små barnens sjukdom och är associerad med dålig nutrition och dålig hygien. Ytterligare predisponerande faktorer är A-vitaminbrist och samtidiga andra infektioner som trakom och mässling (6,7).

Infektiösa keratiter i tropiska länder är oftare bilaterala och leder därmed oftare till blindhet. I östra delarna av Afrika orsakas 70 % av blindhet hos barn av korneala orsaker (ffa infektiösa keratiter) medan motsvarande siffra i Sverige och USA är 1% eller lägre (7,8).

Incidens

Svenska siffror på den årliga incidensen saknas. En populationsstudie från Minnesota, USA, beskriver incidensen av infektiösa keratiter i en begränsad befolkning (Olmsted County) mellan åren 1950 och 1988 (9). Mellan åren 1950 och 1959 var incidensen 2,5 fall per 100 000 invånare och år och ökade till 11 fall per 100 000 invånare och år under perioden 1980-1988. Ökningen var parallell med ökad användning av kontaktlinser. Kontaktlinsbärande var den vanligaste riskfaktorn även under åren 1950-1969, dvs före introduktionen av mjuka kontaklinser. Kontaktlinsassocierade keratiter ökade från 0% av alla keratiter under tiden 1950-1959 till 52 % under tiden 1980-1988. Motsvarande siffra från Storbritannien år 1991 var 65 % (10). Kontaktlinsassocierad keratit drabbade oftare yngre personer (medianålder 28 år), medan keratiter associerad med annan ögonpatologi, såsom t.ex. trauma eller bullös keratopati, oftare drabbade äldre (medianålder 43 resp. 53 år) (9). Man kan anta att en liknande ökning skett i alla länder där kontaktlinsbärande är vanligt. Omräknat till den svenska befolkningen skulle ovanstående incidenssiffror innebära totalt ca 950 fall årligen i Sverige. En svensk studie som ägnas åt incidensen av infektiösa keratiter enbart hos personer som bär kosmetiska kontaktlinser rapporterar en årlig incidens på 2,3 fall per 10 000 bärare (11). Detta skulle innebära ca 120 fall per år bland svenska kontaktlinsbärare. Om man antar att dessa utgör ca 52 % av alla keratiter skulle den årliga svenska incidensen av mikrobiell keratit av alla orsaker ligga på en betydlig lägre siffra, ca 250 fall. Troligtvis är dock siffran för totala antalet kontaktlinsassocierade keratiter i Sverige något större eftersom bärare av bandagelinser och linser för afakikorrektion inte är med i detta svenska material. Dessa grupper löper en högre risk att drabbas av infektiös keratit jämfört med bärare av kosmetiska kontaktlinser (12, 13).

Relativ risk för infektiös keratit hos kontaktlinsbärare jämfört med personer utan någon känd riskfaktor har i en brittisk studie från början av 90-talet beräknats till ca 80. För ögon utsatta för trauma respektive annan

epitelpåverkan (ocular surface disease) var motsvarande värden 14 respektive 7 med 95% konfidensintervall (10).

I gruppen kontaktlinsbärare är det personer som bär mjuka linser dag och natt, s.k. dygnet-runt linser, som löper störst risk. Individer med stabila gaspermeabla linser har lägst risk (10,11). En riskökning med ca 10-20 ggr finns rapporterad vid jämförelse mellan dessa båda linstyper (10,11,14,15). Sk månadslinser (mjuka linser som slängs efter 1 månads användning) har inte säkert inneburit någon minskad risk utan snarare medfört ökad risk, speciellt om denna linstyp inte desinficeras regelbundet (16, 17). Siffror på incidensen vid bärande av s.k. endagslinser eller den senaste typen av syregenomsläppliga dygnet-runt linser, sk silikonhydrogelélinser, finns ännu inte.

Incidensen av amöbakeratit beräknades i USA under åren 1985-1987 till ca 2/1 000 000 kontaklinsbärare (18). I Storbritannien gav en motsvarande beräkning under senare hälften av 1990-talet en 50-faldigt högre siffra (1/ 10 000 bärare!) . Acanthamöba ansågs också vara den vanligaste orsaken till kontaktlinsassocierad keratit i Storbritannien under dessa år (19). En brittisk studie har, som väntat, övertygande visat högst relativ risk för amöbakeratit hos personer som inte alls använder desinfektionsmedel. Det föreligger även en tydligt ökad risk vid användning av desinfektionsmedel med dålig effekt mot amöbacystor som klorbaserade system (17). I slutet av 90-talet har man sett en trend mot minskad förekomst av kontaktlinsassocierad amöbakeratit. En populationsstudie i Storbritannien mellan åren 1992 och 1996 har visat en påtagligt minskad incidens under 1996 jämfört med de närmast föregående åren med en halvering av antalet fall (20). I Sverige kom de första odlingsverifierade fallen i början av 90-talet med en smärre epidemi omfattande 8 fall i södra delarna av landet under åren 1991-1993 (21). Den årliga incidensen därefter är uppskattningsvis 1 fall per år i hela landet (Skarin, opubl.). Minskningen av incidensen under senare hälften av 90-talet tillskrivs förbättrade hygieniska rutiner bland kontaktlinsbärare (20).

Etiologi och patogenes

Etiologi

Bakterier

De flesta kända humanpatogena bakterierna anses kunna orsaka keratit (1, 4). Det vanligast odlingsfyndet i tempererade klimatzoner är Grampositiva kocker ffa stafylokocker medan Gramnegativa organismer ffa *Pseudomonas aeruginosa* är vanligare i odlingar från patienter i tropiska klimatzoner (2,22,23,24,25,26,27,28).

*Tabell 1 Odlingsfynd från oselekterade material av keratiter från olika klimatområden. Siffrorna anger procentuella fördelningen i den grupp som varit odlingspositiv. (n= totala antalet odlingspositiva) * även blandfloror medräknade varför summan överstiger 100 %/ * i denna studie anges enbart Genusnamnet* Pseudomonas

Agens	Göteborg 1987-1990 (Neumann, Sjöstrand 1992) n=30	Baltimore 1985-1989 (Whale et al 1991) n=58	Californien* 1972-1983 (Ormerod et al 1987) n=186	Florida 1969-1977 (Liesegang et al 1980) n=371	Bangladesh (1990-tal ?) (Dunlop et al1994) n=117	Ghana (1990-tal?) Hagan et al 1995 n=114
Stafylokocker						
S. epidermidis	30	28	29	5	0	11
S. aureus	30	16	22	14	2	3
Streptokocker						
S. Pneumoniae	13	12	15	5	20	6
övriga Strept.	2	14	19	6	1	3
Moraxella	3	2	8	1	1	3
Pseudomonas	10					
P. aeruginosa	/ *	16	19	20	15	12
övriga Gramnegativa stavar	12	2	17	9	17	4
Svamp	0	2	13	36	40	51
övrigt	0	9	5	4	2	6

Bakteriella keratiter hos kontaktlinsbärare i tempererade klimatzoner orsakas i upp till 40 % fallen av Gramnegativa bakterier, ffa *Pseudomonas aeruginosa*. (2,19,28,29,30,31,32).

Tabell 2.

Odlingsfynd från keratiter associerade med kontaklinsbärande. Siffrorna anger den procentuella fördelningen i gruppen av odlingspositiva.
n= totala antal odlingspositiva
**=fynd från blandflora medräknat varför summan överstiger 100%*
*/ * I dessa studier anges enbart Genusnamnet* Pseudomonas

Agens	Göteborg* 1987-1990 (Neumann, Sjöstrand 1992) n=10	Baltimore 1985-1989 (Whale et al 1991) n=22	Philadelphia 1983-1984 (Laibson, Donnenfeld 1986) n=34	Philadelphia 1988-1995 (Cohen et al 1996) n=143
Stafylokocker			11	25
S. epidermidis	50	25	-	-
S. aureus	20	8	-	-
Streptokocker			15	
S. Pneumoniae		13	-	
övriga Strept.		17	-	
Moraxella		4		
Pseudomonas	20		58	37
P. aeruginosa	/ *	29	/ *	/ *
övriga Gramnegativa stavar	20		15	
Svamp				
Acanthamöba				11
övrigt		4		27

Keratiter orsakade av anaeroba bakterier är beskrivet i litteraturen. Incidensen rapporteras lägre än för de vanliga fakultativt anaeroba arter och är osäker eftersom anaerob odling inte alltid utförs rutinmässigt vid keratitodlingar (33,34,35,36).

Svamp

Dessa infektioner är vanligast i varma klimatzoner och relativt sällsynta i tempererade (Tabell 1). Från Bangladesh har en frekvens på 40 % rapporterats medan den i norra USA och Europa är ca 2 % eller lägre (2,22,28) .Klimatfaktorer påverkar vilket infekterande agens som dominerar; således är *Aspergillus* och *Fusarium* dominerande i varma torra

klimatzoner medan de är mindre vanliga i varmt fuktigt klimat .Jästsvampar såsom *Candida* är vanligast i tempererade klimatzoner (2,22,23,27).

Protozoer

Acanthamöbakeratit var fram till mitten av 80-talet mycket sällsynt och rapporterades i västerländsk litteratur dessförinnan som sporadiska fall associerade till trauma eller systemisk infektion (36,37). Därefter ökade antalet fall dramatisk och en stark association sågs till kontaklinsbärande ffa av mjuka linser. Amöban överförs sannolikt främst från kontaminerat vatten t.ex. kranvatten (18,19).

Polymikrobiella keratiter

Polymikrobiella keratiter rapporteras i vissa material utgöra ca 15% av keratiterna och förekommer ffa. hos barn och immunsupprimerade patienter (24,38,39,40) . Både kombinationer av olika bakteriearter, aeroba och anaeroba, och kombinationer av svamp och bakterier förekommer (41) . Bakteriell superinfektion med ffa streptokockarter, i ögon med pågående Acanthamöbakeratit finns också beskrivet (19,21).

Patogenes

Kornea exponeras ständigt för mikroorganismer från ögonlock, fingrar och kontaminerade partiklar i luft, vatten och annat som kommer i kontakt med tårfilmen. Dessa organismer elimineras effektivt med hjälp av både ospecifika och specifika skyddsmekanismer. Till de ospecifika räknas dels rent mekaniska faktorer som tårvätskans ständiga nybildning och avflöde vilket befrämjas av normal blinkfunktion samt epitelets ständiga nybildning. Till det ospecifika immunsvaret räknas ämnen och celler i tårfilmen som har direkt hämmande effekt på mikroorganismernas överlevnad såsom, lysozym, lactoferrin, komplementfaktorer, makrofager, NK-celler och granulocyter. Dessa samverkar integrerat och styrs av det specifika immunförsvaret representerat av immunglobuliner ffa Ig A och Ig G samt cytokiner från T- och B-lymfocyter (42,43).

Konjunktivan har samma typ av immunologisk övervakning som kroppens övriga slemhinnor s.k. MALT (mucosa associated lymphoid tissue). Lymfa dräneras från de mediala delarna till submandibulära lymfkörtlar och från laterala till preaurikulära och till körtlar i parotis. Vävnadsbundna makrofager och andra antigenpresenterande celler t.ex. Langerhans celler eller B-lymfocyter kan ta upp främmande antigen, interagera med T-lymfocyter och därmed starta en aktivering av det specifika immunförsvaret vilket i sin tur dirigerar och effektiviserar t.ex. komplementfaktorer och granulocyter (43).

Den vaskulariserade delen av perifer kornea övervakas främst av konjunktivans immunsystem. Där finns granulocyter av alla slag liksom T-

och B-lymfocyter samt mastceller. I korneas perifera delar finns också antigenpresenterande Langerhanska celler, vilka saknas centralt på kornea. Lösliga ämne som komplementfaktorer och immunglobuliner finns i högre koncentration i perifera kornea än i centrala kornea (43).

Endast ett fåtal mikroorganismer tros på egen hand kunna forcera intakt och friskt kornealepitel. *Neisseria* och *Acinetobacter* är dokumenterade i litteraturen sedan 60-talet (44,45) medan *Listeria*, *Shigella*, *Haemophilus* och *Corynebacterium diphteriae* anges anekdotiskt (1,4). Adhesionsmolekyler som pili och adhesiner som kan inducera aktiv fagocytos hos epitelcellerna, epitelskadande toxiner samt det faktum att t ex Neisseriaarterna orsakar en massiv rekrytering av neutrofila leukocyter som i sin tur secernerar lytiska enzym tros vara främsta orsakerna (44,46,47). Övriga potentiellt farliga mikroorganismer anses enbart kunna etablera en infektion under epitelet om det finns en försämrad epitelbarriär t ex. en epitelskada (1,4).

Patofysiologi

Riskfaktorer allmänt

Tillstånd som nedsätter det immunologiska skyddet och/eller ökar exponeringen för mikroorganismer medför ökad risk för infektiös keratit. Den faktor som i västvärlden främst förknippas med infektiös keratit är kontaktlinsanvändning. Desensibilisering av kornea, nedsatt blinkfunktion, reducerad tårproduktion, dakryocystit, sjukligt förändrat epitel t ex vid kornealödem är andra (9,10,48). Även iatrogena faktorer som användning av kortisondroppar och korneal kirurgi ökar risken (24). Medvetslöshet, diabetes, psykiatrisk anamnes liksom rökning och alkoholism och etnisk tillhörighet har också visat association till ökad risk för infektiös keratit (14,23).

De patofysiologiska mekanismerna vid infektiös keratit associerad med kontaklinsanvändning förefaller vara multipla. Kornea utsätts troligtvis för en ökad exponering för mikroorganismer genom att:

1)försämrad elimination av mikroorganismer orsakat av att kontaktlinsen mekaniskt påverkar tårvätskans sköljning av korneas yta. 2) Kontaminerade kontaklinsvätskor medföra en direkt inokulation av potentiellt patogena organismer. 3) Mikroorganismer kan binda till biofilm på kontaklinsen eller till själva linsmaterialet (19,49) .

Korneas immunförsvar reduceras av den hypoxi som uppträder då kontaktlinsen är placerad över kornea många timmar i sträck (50). Dessutom kan insättning och utplockning av linsen innebära en risk för epitelskada.

Eftersom bärandet av dygnet-runt linser förknippas med störst risk för infektiös keratit torde försämrad elimination av kontaminerande organismer och epitelpåverkan vara mycket betydelsefulla vid uppkomsten av infektionen.

Riskfaktorer svampkeratit

Varmt torrt klimat samt ögontrauma i jordbruksmiljö medför högst risk för att drabbas av svampkeratit (22,27). De vanligast organismer är i detta sammanhang Aspergillus- och Fusariumarter medan Candida eller andra jästsvampar dominerar vid de få svampkeratiter som uppträder i tempererade klimatzoner (2). Riskfaktorer som epitelsjukdom , kontaklinsbärande och lokal eller systemisk immunsupression är också aktuella för svampkeratiter (22).

Riskfaktorer Acanthamöbakeratit

Före mitten av 1980-talet var trauma den största riskfaktorn vid Acanthamöbakeratit (18,36,37). Därefter uppträdde en formlig epidemi i USA och Västeuropa med fall associerade till kontaktlinsbärande. Inkorrekta rengöringsrutiner och t ex användning av osteril hemmablandad koksaltlösning befanns starkt associerad till infektionen i USA (18,51). Brittiska studier har sett liknande samband med felaktiga desinfektionsrutiner ffa utebliven desinfektion samt bruk av klorbaserade desinfektionsmedel (17). Bruk av s.k. månadslinser (i engelsk litteratur kallade ”disposable lenses”) var också associerat med en ökad risk. Detta har inte uppenbart varit fallet bland de få fall som hittills uppträtt i Sverige (21).

Symtom och klinisk bild

De subjektiva symtomen är vanligtvis fotofobi, varierande grad av smärta, varierande grad av synnedsättning, tårflöde och eventuellt purulent sekret.

De vanligaste objektiva symtomen är 1) monokulär konjunktival injektion av ciliär typ. 2)På kornea ses vanligtvis en härd med epitelskada och omgivande epitelialt ödem samt ödem och varierande grad av destruktion av kornealt stroma med omgivande ödem (Fig. 1 och 2). 3) Då korneas stroma engageras uppträder inflammatoriska celler i främre kammaren. Infektionen progredierar från ett ingångsställe i epitelet och vidare i stromal vävnad åt sidorna och på djupet. Infektioner orsakade av Nocardia, svamp, Mycobakterier och *Acanthamöba* debuterar oftast stillsamt i form av progredierande epiteliopati som till utseendet kan påminna om recidiverande herpeskeratit s.k. "dendriticakeratit " (52,53,54). Vid debuten av en Acanthamöbakeratit har patienten ofta intensiva smärtor som kontrasterar till de diskreta fynden på kornea. Acanthamöbakeratit är oftast lokaliserad centralt och symmetrisk på kornea.

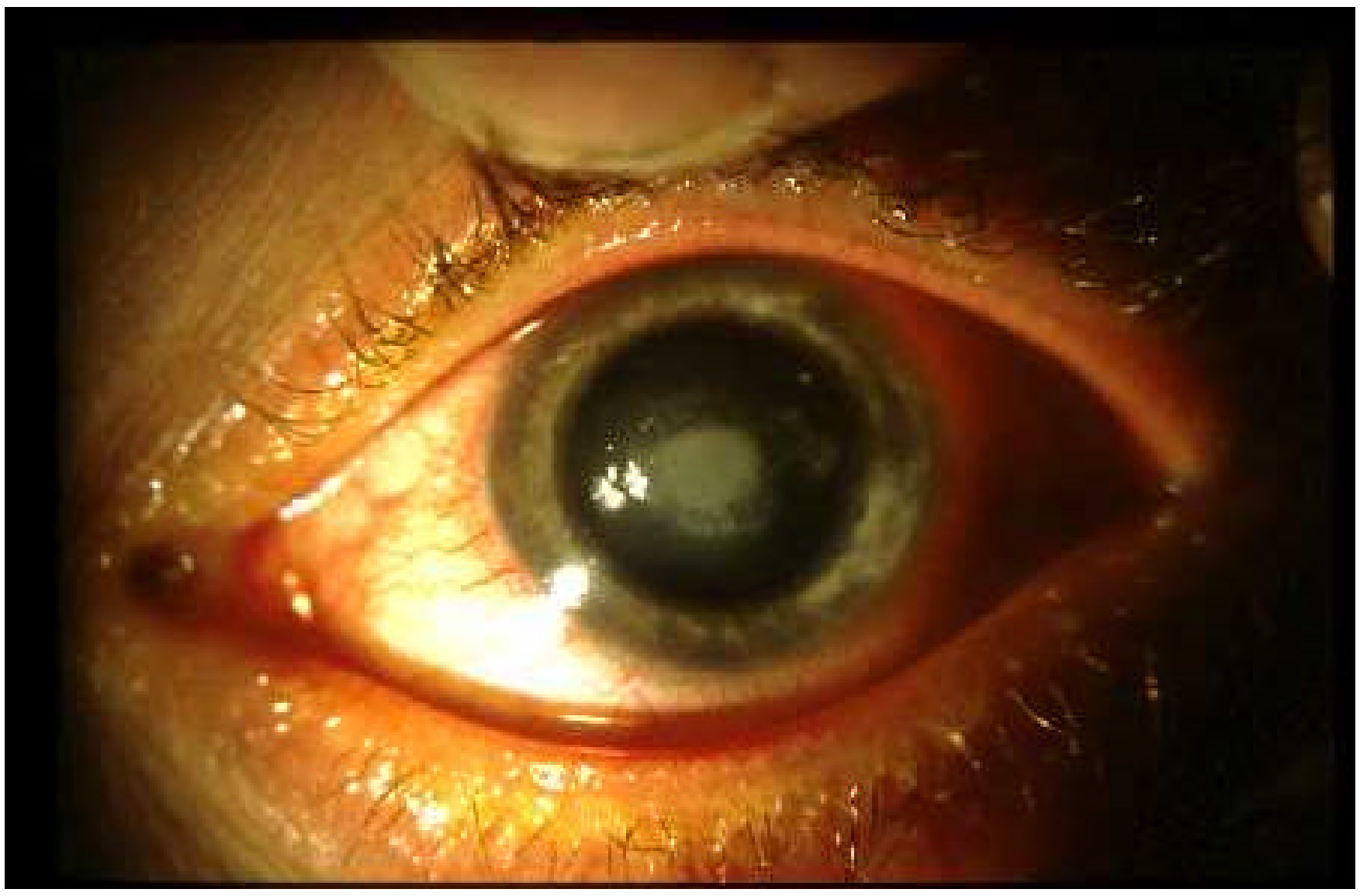

Figur. 3. Central Acanthamöbakeratit

Infektioner som utvecklas i samband med pågående kortisonbehandling t. ex efter korneal kirurgi, kan också ha ett stillsamt förlopp, s.k. interstitiell keratit utan kornealt ödem där organismerna växer infiltrativt i stromat med intakt epitel över.

Neisseriaarter ger kraftig samtidig purulent konjunktivit och kan snabbt progredierar till keratit och risk för perforation. *Pseudomonas aeruginosa*-keratit (Fig. 4 och 5) karakteriseras också av hastiga förlopp med snabb spridning (timmar-dagar) över hela kornea och stor risk för perforation. Stafylokockkeratiter har vanligen något långsammare förlopp (Fig. 2) (1,4).

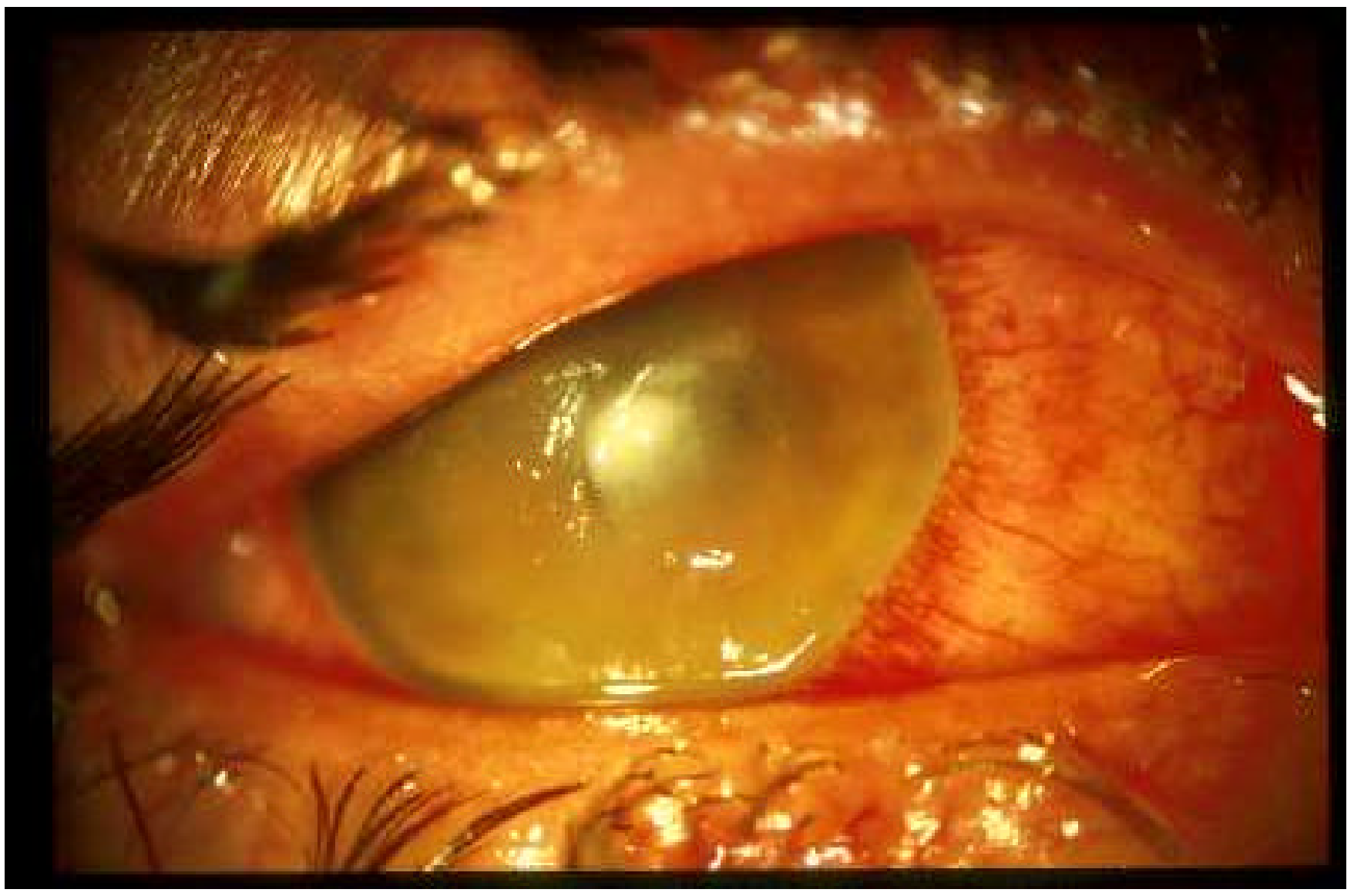

Figur 4. Akut keratit orsakad av P Aeruginosa

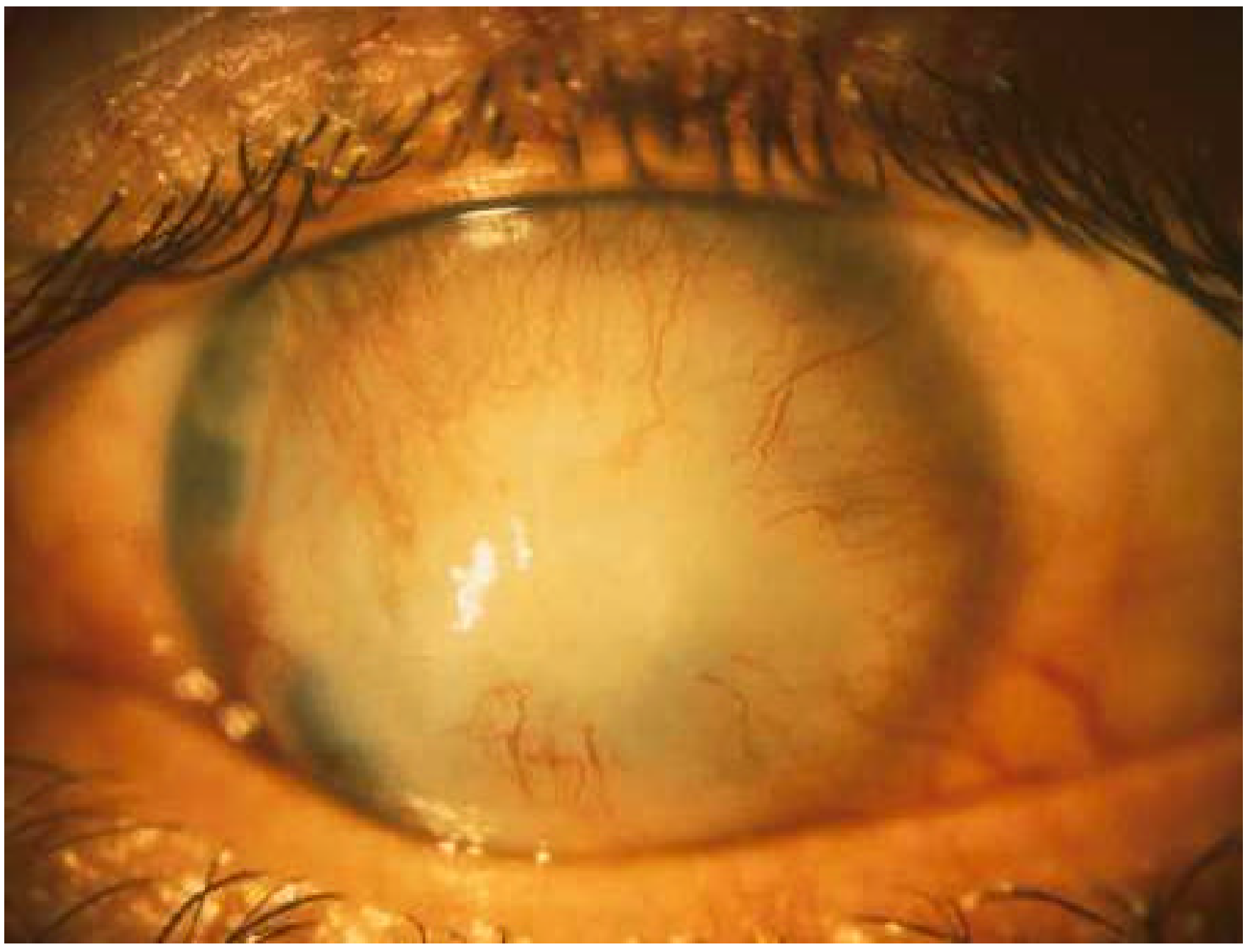

Figur 5. Utläkt keratit orsakad av P.aeruginosa, vit kornea

Utredning/diagnostik

Odling

Sedan 70-talet rekommenderas odling från den infekterade härden. (1,55). En diskussion pågår angående nyttan av odling och resistensbestämning, men ingen har ännu vågat ge rekommendationer om när man riskfritt kan avstå från provtagning (56,57,58).

Pga små provvolymer och därmed lågt antal organismer är det en fördel om materialet inokuleras direkt på odlingsmedier, detta för att undvika den utspädning av provet som inokulation på ett transportmedium innebär.

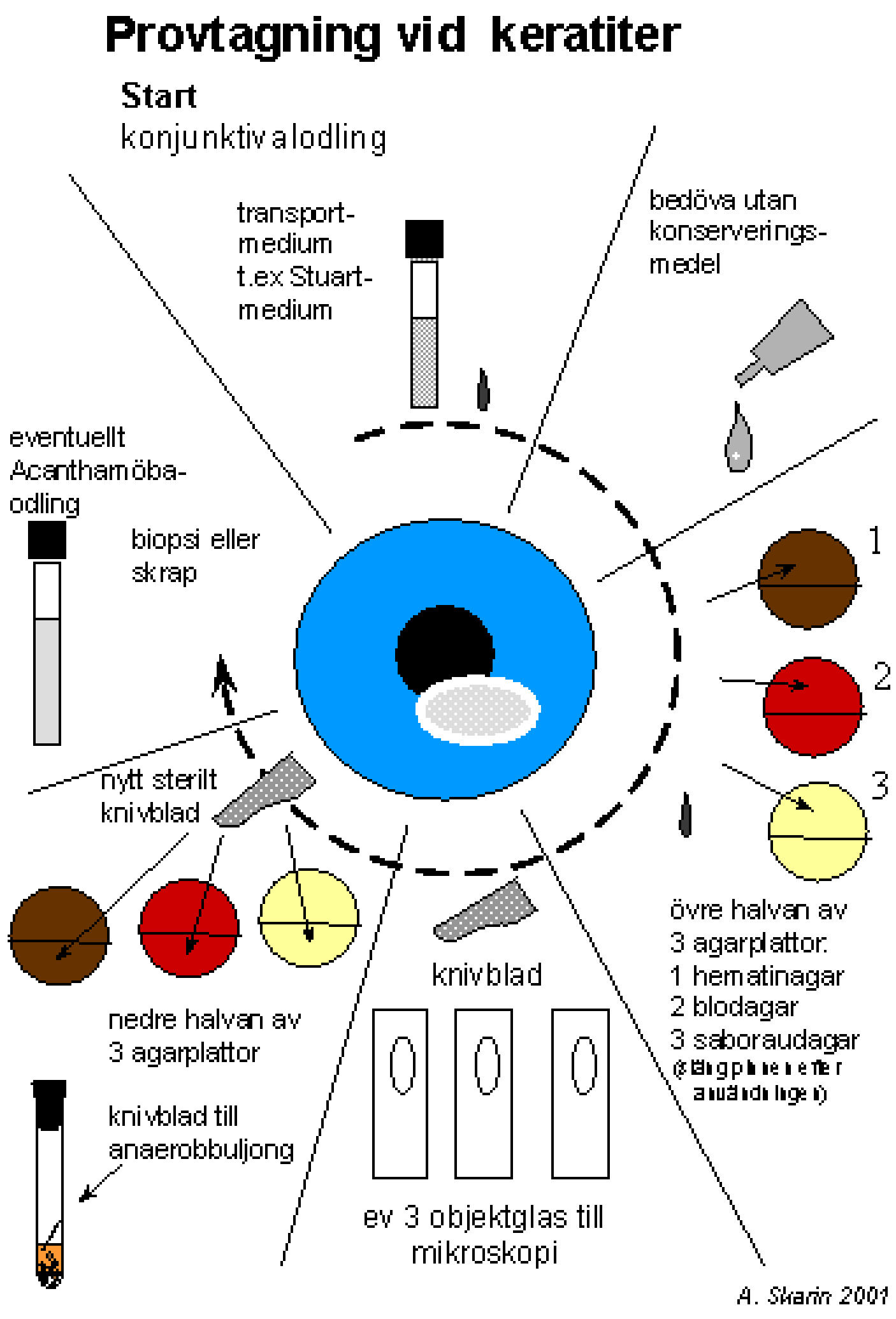

Figur. 6. Odlingsschema

Detta odlingsförfarande förutsätter dock kort transporttid (enstaka timmar) till laboratoriet. Konjunktivan kan även hos friska personer ha en låg grad av bakterieförekomst vilket försvårar tolkningen av odlingsresultatet. Om provet inokuleras på flera olika medier kan den sammanlagda bilden från

dessa underlätta tolkningen. Provet bör tas vid kanten av keratithärden med sterilt instrument som tillåter att man skrapar kornea och som inte skadar ytan på agarmediet vid inokulation. Ett smalt sterilt knivblad som hålls med en steril peang är utmärkt

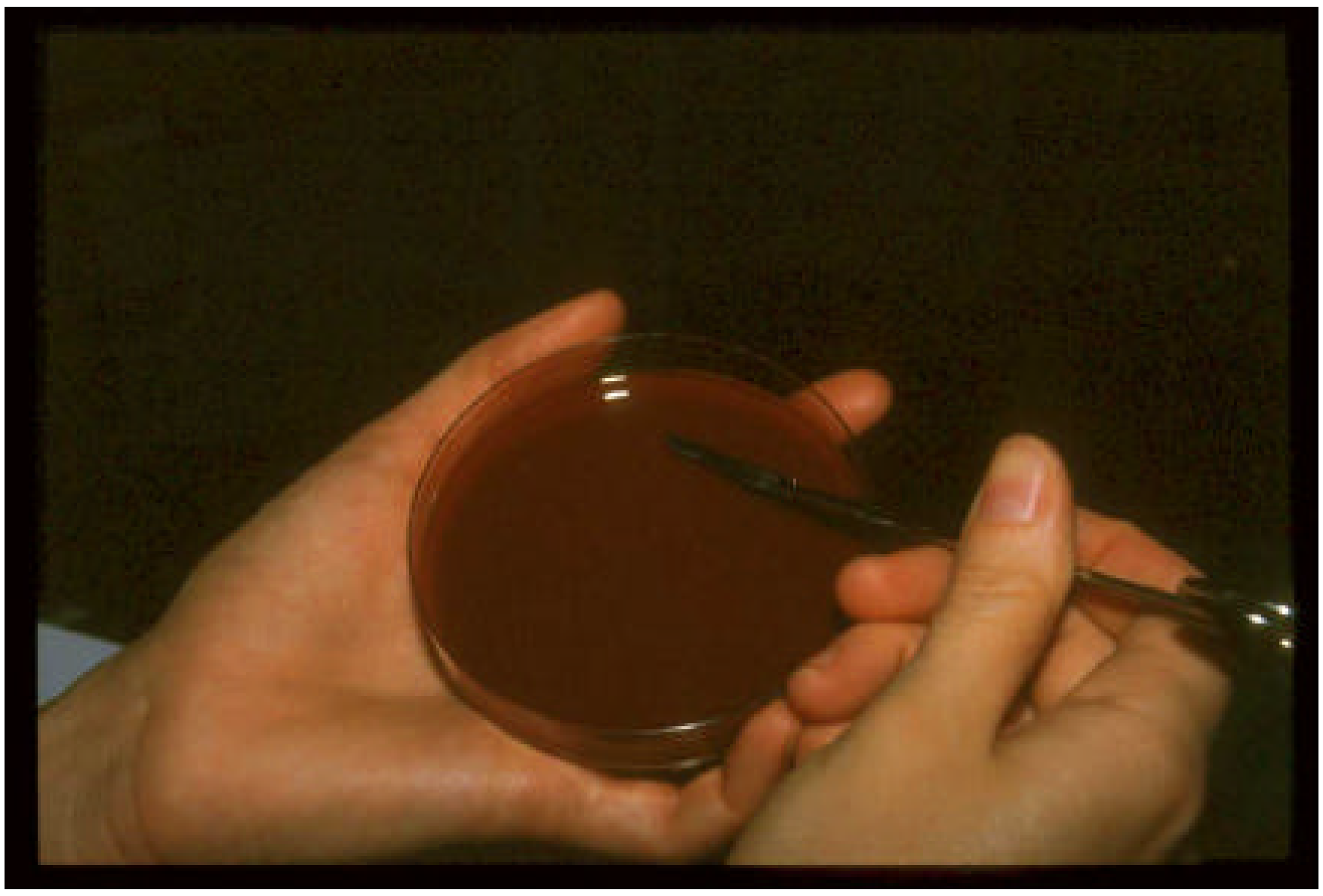

Figur. 7. knivblad hållet med steril peang och hematinagar

Pus och nekrotiska celler över härden skall först avlägsnas och kan eventuellt också skickas till odling. Om samma organism (eller organismer) påträffas på två eller flera odlingsmedier talar detta för att den orsakar keratiten (24). Eftersom vissa organismer enbart kan odlas in vitro under speciella betingelser, och därmed inte kan växa fram på mer än ett av medierna bör en klinisk bakteriolog kontaktas vid svårtolkade odlingsfynd. Om flera arter odlas fram i låga kvantiteter med olika bakterier på varje medium kan detta tala för att provet blivit kontaminerat från t ex ögonlockshud. Polymikrobiella keratiter förekommer dock men för att ställa den diagnosen skall odlingen helst visa samma polymikrobiella bild på flera odlingsmedier (24,38,39,40).

Vid uteblivet positivt odlingsfynd och progredierande keratit rekommenderas utsättning av pågående behandling i helst 2 dygn, varefter förnyad provtagning görs med utvidgade frågeställningar t.ex. svamp, mycobakterier och Nokardia (59). Prov för mikroskopisk undersökning kan i detta läge vara mycket värdefulla (8) (se Mikroskopi).

Mikroskopi

Att ta prov för Gramfärgning (som skiljer mellan Grampositiva och Gramnegativa bakterier), acridinorange (alla bakterier), Ziehl- Nielsen (mycobakterier och Nocardia), silverfärgning (vissa svampar) och färgning med hjälp av fluorescerande mono- eller polyklonala antikroppar rekommenderas generellt i amerikansk och brittisk litteratur (19,24,55). I

Sverige används oftast Gram- och eventuellt metylenblåfärgning. Övriga tekniker utnyttjas enbart vid specialfrågeställningar. Mikroskopi är värdefullt ffa vid svampkeratiter eller odlingsnegativa keratiter då det kan röra sig om svårodlade organismer som inte växer på de medier som används rutinmässigt (8).

I ett Gramfärgat preparat kan organismen identifieras i ca 75 % av fallen vid infektion med enstaka bakterier och i 37% av fallen vid polymikrobiella infektioner (38). Som jämförande facit används i denna publikation odlingsresultatet vilket antyder att utgångspunkten är att färgning har betydligt lägre specificitet och sensitivitet än en odling. Mikroskopisk undersökning kan f.n. inte jämställas med eller ersätta en odling. Konfokalmikroskopi har använts vid diagnostik av odlingsnegativa misstänkta Acanthamöbainfektioner men kan idag i praktiken inte ersätta traditionella tekniker (60).

Känsligheten kan ökas med hjälp av monoklonala antikroppar eller PCR-teknik men dessa tekniker förutsätter att man i förväg måste bestämma vilken organism som skall detekteras. Risken för falskt positiva resultat från prover på kornea är uppenbar vid försök till detektion av organismer som förekommer i normalfloran. PCR-tekniken förefaller mest attraktivt vid detektion av sällsynta och/eller svårodlade organismer (61,62).

Behandling

Naturalförlopp

Obehandlat kan ett infekterat kornealsår leda till blindhet pga perforation med sekundär endoftalmit eller pga kraftig ärrbildning på kornea. Infektionens progress är beroende av den infekterande organismens virulens (se etiologi) och det infekterade ögats förmåga till immunsvar. Dålig blinkförmåga, nedsatt tårproduktion, desensibilisering av kornea eller annan sjuklighet i kornea och eller konjunktiva medför sämre förutsättningar för läkning.

Medikamentell behandlig av bakteriella keratiter

Tabell 3

Preparat mot Grampositiva bakterier	(mg/ml)	kommentar
ciprofloxacin (Ciloxan ™)	3	
vancomycin	50	
Penicillin G	100 000	(E/ml)
cefuroxim	50	
amikacin	25-50	
kloramfenikol (Kloramfenikol ™)	5	
fucidinsyra (Fucithalmic ™)	10	salva (mg/g)
klindamycin	15	

Tabell 4

Preparat mot Gramnegativa bakterier	(mg/ml)	kommentar
cefuroxim	50	dock ej P. aeruginosa
amikacin	25-50	
gentamicin	15	svider
tobramycin	15	
kloramfenikol	5	dock ej P. aeruginosa
ciprofloxacin (Ciloxan ™)	3	

Principen för antibiotikabehandling är att initialt droppa intensivt minst 1 gång i timmen med koncentrerade antibiotikadroppar som har effekt mot både Grampositiva och Gramnegativa organismer. I praktiken innebär det vanligtvis att två olika preparat ges parallellt, t. ex . klindamycin eller cefuroxim och en aminoglykosid (gentamicin eller tobramycin). Fluoroquinolon-preparat (ciprofloxazin, norfloxacin ofloxacin) som på

senare år blivit kommersiellt tillgängliga som ögondroppar har använts framgångsrikt i monoterapi (64,65). Preparaten har främst effekt mot Gramnegativa bakterier inklusive Pseudomonas aeruginosa medan Stafylokocker har rapporterats mindre känsliga. I klinisk praxis har man ännu inte sett tecken på terapisvikt mot stafylokocker men den behandlande ögonläkaren bör vara medveten om risken, speciellt med tanke på att Stafylokocker är vanligt etiologiskt agens inom vår klimatzon (se etiologi). De standardiserade resistensbestämningar som utförs på kliniskt mikrobiologiska laboratorier är anpassade till peroral eller intravenös behandling med preparaten. De koncentrationer som uppnås vid lokal droppning i ögat överstiger de terapeutiska nivåerna som erhålls vid peroral och intravenös terapi. Detta medför att en substans mycket väl kan ha god antibakteriell effekt vid keratitbehandling trots att resistensbestämningen anger resistens hos den testade bakterien (66). Kliniskt terapisvar måste vara vägledande för den fortsatta antibakteriella behandlingen och en diskussion med den kliniska bakteriologen är alltid värdefull.

Medikamentell behandling av svampkeratiter

Tabell 5.

Preparat mot svamp	(mg/ml)
Natamycin	50
Amfotericin B	1,5-3
Fluconazol	10-20
Miconazol	10
Clotrimazol	10

Pga att svampkeratit är relativt sällsynt i Sverige sätts antimykotisk behandling in först när odlingen och/eller mikroskopi uteslutit bakterier och påvisat förekomst av svamp. Resistensbestämning görs inte rutinmässigt vid fynd av svampar varför behandlingen med fördel bör diskuteras med infektionsläkare eller mykolog. I Sverige finns inga kommersiellt tillgängliga ögondroppar för behandling av svampkeratit varför dessa får förskrivas på licens och eventuellt blandas extempore. Natamycin finns i USA (Natacyn™) och är ffa. effektivt mot Aspergillus- och Fusariumarter. Amfotericin B är effektivast mot jästsvampar såsom Candida men har känd korneal toxicitet. Imidazolpreparat som miconazol och ketoconazol kan ha effekt mot både jästsvampar och filamentösa svampar. (19,52,67)

Medikamentell behandling av Acanthamöbakeratit

Tabell 6

mot Acanthamöba	mg/ ml	Kommentar
Propamidin isethionat (Brolene ™)	1	
Hexamidin isethionat	1	
Klorhexidin diglukonat	0,2	
Polyhexametylen biguanid	0,2	PHMB
Clotrimazol	10	
Paromomycin	15	

Denna infektion behandlas som regel med minst två preparat samtidigt. Propamidine isethionate (Brolene™) är det som vanligast används kombinerat med t. ex. klorhexidin diglukonat eller polyhexametylene biguanid (PHMB). Hexamidin isethionat (Desmodin™) är ett alternativ till propamidin isethionat. Också paromomycin, clotrimazol, micotazol, fluconazole, ketoconazole och itraconazole har använts främst som tilläggsbehandling. Inga av dessa preparat är tillgängliga i Sverige varför licensförskrivning och ex-temporeberedning är regel (4, 18,63,64) Peroral terapi rekommenderas ffa i amerikansk litteratur (4,18, 64). den lokala behandlingen ges en gång i timman de första dygnen med mycket långsam nedtrappning under loppet av flera månader beroende på terapisvar och eventuella toxiska effekter av dropparna.

Från ca 1 vecka till 1 månads behandling uppträder i vissa fall en korneal reaktion i form av ökande ödem och ökad mängd inflammatoriska celler i främre kammaren. Denna kliniska försämring kan lätt misstolkas som progress av infektionen men anses vara en inflammatorisk reaktion mot frisläppta amöbaantigen. Tillståndet kan vara svårbehandlat (19,63,64). I klinisk praxis ges ofta ett tillägg av kortisondroppar (64). Tidig diagnos och därmed tidig behandling har de senaste åren förbättrat prognosen för denna svåra infektion (18,19,20,21,63,64).

Kirurgisk behandling

Tarsorafi är i de flesta fall nödvändigt för läkning av keratiter i ögon med desensibiliserad kornea, nedsatt tårproduktion, felställningar i ögonlock eller andra faktorer som kraftigt försämrar läkningsförmågan. Om nedsmältningen av kornea blir så uttalad att perforation hotar eller uppstår kan akut kirurgi försökas; En komplett eller partiell konjunktival lambå kan dras ner över infektionshärden. Man kan göra en limbusbaserad lambå som viks ner med epitelsidan mot kornea. Vid misstänkt aktiv infektion är dock en blodförande konjunktival lambå att föredra. Vävnads lim (Histacryl, Braun Melsungen AG) är en tillfällig åtgärd som efter någon vecka till månad kräver definitiv kirurgisk korrektion. Amnionhinna kan också

utnyttjas. I själva såret får hinnan packas i flera lager och kan förankras med fibrinlim (Tisseel, Immuno) för att slutligen täckas av en hinna över hela ögats yta som sutureras(68). Akut tektonisk, penetrerande keratoplastik kan behövas, men detta är förknippat med stor komplikationsrisk och skall helst inte övervägas förrän den akuta infektionen är säkert under kontroll och helst utläkt (63). Vid keratiter orsakade av långsamväxande och svårbehandlade organismer såsom svamp och acanthamöba kan det vara omöjligt att avgöra när infektionen är utläkt. Man skall vara medveten om att det kirurgiska ingreppet kan medföra att infektionen blossar upp och sprids intraokulärt. I stället för penetrerande transplantation kan lamellär transplantation ibland rädda situationen.

Botulinustoxininjektion i levatormuskeln som ger en iatrogen ptos i ca 4 veckor kan i vissa fall vara användbart i de fall en tarsorafi inte är nödvändigt t ex då en stor epiteldefekt kvarstår efter en utläkt keratit (69). I det akuta skedet bör detta dock undvikas eftersom en total slutning av ögonlocket kan gynna infektionen.

Specifik medikamentell behandling skall helst påbörjas före eventuella kirurgiska ingrepp och fortsätta så länge som infektionen bedöms pågå.

Vid rekonstruktion av kornea efter en keratit skall ljusspridande ärrvävnad ersättas med klar transparent och en ojämn hornhinneyta ersättas med en jämn optiskt fungerande yta. Excimerlaserkirurgi kan utnyttjas till bägge dessa åtgärder förutsatt att grumlingen är ytlig och att ojämnheten engagerar den främre ytan. Lamellär keratoplastik är ibland ett alternativ liksom penetrerande keratoplastik. Den sistnämnda kan göras som autograft i form av en rotationsplastik emellanåt alternativt som ett allograft om grumlingar och ojämnheter är djupt belägna.

Referenser

1. O´Brien TP.Bacterial keratitis, in Cornea eds. Krachmer JH, Mannis MJ, Holland EJ. 1997 Mosby-Year Bood Inc. 1139-89

2. Whale JC, Katz HA, Abrams DA. Infectious keratitis in Baltimore. Ann Ophthalmol. 1991; 23: 234- 7.

3. Referensmetodik för laboratoriediagnostik vid klinisk mikrobiologiska laboratorier. I. Infektionsdiagnostik. 7 Ögoninfektioner. Smittskyddsinstitutet 1994

4. Wilhelmus K R Bacterial keratits in Ocular Infecction & Immunology. Eds. Peposo JS, Holland GN, Willhelmus KR. Moseby -Year Book Inc. 1996: 970-1031.

5. Seal DV, Ficker LA, Wright P. Staphylococcal blepharitis. in Ocular Infecction & Immunology. Eds. Peposo JS, Holland GN, Willhelmus KR. Moseby -Year Book Inc. 1996: 788-98.

6. Foster A, Yorston D. Corneal ulceration in Tanzanian children: relationship between measles and vitamin A deficiency. Trans R Soc Trop Med Hyg. 1992; 86: 454-455.

7. Foster A, Gilbert. Epidemiology of childhood blindness. Eye. 1992;6: 173-6.

8. Blohme´J, Tornqvist K. Visual impairment in Swedish children. II Etiological factors. Acta Ophthalmol Scand. 1997; 75: 199-205.

9. Erie JC, Nevitt MD, Hodge DO, Ballard DJ. Incidence of ulcerative keratits in a defined population from 1950 through 1988. Arch Ophthalmol. 1993; 111:1665-71.

10. Dart KG, Stapelton F, Minassian. Contact lenses and other risk factors in microbial keratitis. Lancet. 1991; 338: 650-3

11. Nilsson SE, Montan PG. The annualized incidence of contact lens induced keratitis in Sweden and its relation to lens type and wearing schedule: Results of a 3-month prospective study. The CLAO J. 1994;20: 225-30.

12. Kent HD, Cohen EJ, Labison PR, Arentsen JJ. Microbial keratitis and corneal ulceration associated with therapeutic soft contact lenses. The CLAO J. 1990; 16: 49-52.

13. Glynn RJ, Schein OD, Seddon JM et al. The incidence of ulcerative keratitis among aphacia contact lens wearers in New England. Arch Ophthalmol; 109: 104-7.

14. Schein OD, Poggio EC. Ulcerative keratitis in contact lens wear. Incidence and risk factors. Cornea. 1990;9: 555-8.

15. Poggio EC, Glynn RJ, Schein ODSedon JM, Shannon MJ, Scardino V A, Kenyon KR. The incidence of ulcerative keratitis among users of daily-wear and extended-wear soft contact lenses. N Engl J Med 1989; 321: 779-83.

16. Chatterjee A, Kwartz J, Ridgway AEA, Storey JK. Disposable soft contact lens ulcer: A study of 43 cases seen at Manchester Royal Eye Hospital. Cornea. 1995; 14: 138-41.

17. Radford CF, Bacon AS, Dart JKG, Minassian DC. Risk factors for acanthamoeba keratits in contact lens users: a case-control study. Br Med J. 1995; 310: 1567-70

18. Schaumberg DA, Snow KK, Dana MR. The epidemic of Acanthamoeba keratits: Where do we stand? Cornea. 1998; 17: 3- 10.

19. Seal DV, Bron AJ, Hay J. Ocular infection. Martin Dunitz Ltd , UK. 1998, 37-83.

20. Radford CF, Lehman OJ, Dart JK. Acanthamoeba keratits: multicenter survey in England 1992-96.
National Acanthamoeba Keratits Study Group. Br J Ophthalmol. 1998; 82:1387-92

21. Skarin A, Florén I, Kiss K, Miörner H, Stenevi U. Acanthamoeba keratits in the south of Sweden. Acta Ophthalmol Scand 1996; 74: 593-7.

22. Dunlop AAS, Wright ED, Howlader SA, Nazrul I, Husain R, McClellan K, Billson FA. Suppurative corneal ulceration in Bangladesh. Austr New Zee J Ophthalmol. 1994; 22:105- 10.

23. Ormerod LD, Hertzmark E, Gomez DS, Stabiner RG, Schanzlin DJ, Smith RE. Epidemiology of microbial keratitis in southern California. Ophthalmol. 1987;94: 1322-33.

24. Gudmundsson OG, Ormerod LD, Kenyon KR et al. Factors infuencing predilection and outcome in bacterial keratitis. Cornea 1989; 8: 115-21.

25. Hagan M, Wright E, Newman M, Dolin P, Johnson. Causes of suppurative keratitis in Ghana. Br J Ophthalmol. 1995; 79: 1024-8-

26. Jones DB. Early diagnosis and therapy of bacterial corneal ulcers. Int Ophthalmol Clin. 1973;13 (4): 1-29.

27. Lisegang TJ, Forster RK. Spectrum of microbial keratitis in south Florida. Am J Ophthalmol. 1980; 90: 38-47.

28. Neumann M, Sjöstrand J. Central microbial keratitis in a swedish city population. Acta Ophthalmol. 1992; 70: 160-4.

29. Cohen EJ, Fulton JC, Hoffman CJ, Rapuano CJ, Laibson PR. Trends in contatct lens-associated corneal ulcers. Cornea 1996; 15: 566-70.

30. Laibson PR, Donnenfeld ED. Corneal ulcers related to contact lens use. Int Ophth Clin. 1986; 26: 3-14

31. Schein OD, Ormerod LD, Barraquer E, Egan KM, Kenyaon KR. Microbiology of contact lens-related keratitis. Cornea. 1989; 8: 281-5.

32. Stapelton F, Dart JKG, Seal DV et al. Epidemiology of Pseudomonas aeruginosa in contact lens wearers. Epidem Infect. 1995;114: 395- 402.

33. Perry LD, Brinser JH, Kolodner H. Anaerobic corneal ulcers. Ophthalmology. 1982; 89: 636- 42.

34. Stern GA, Stock EL. Experimental Bacteroides fragilis keratitis. 1978; 96: 2264- 6.

35. Stern GA, Hodes BL, Stock EL. Clostridium perfringens corneal ulcer. Arch Ophthalmol. 1979; 97: 661- 3.

36. Jones DB, Visvesvara GS, Robinson NM. Acanthamoeba polyphaga keratits and Acanthamoeba- uveitis associated with fatal meningoencephalitis. Trans Ophthalmol Soc UK. 1975; 93:221- 31.

37. Nagington J, Watson PG, Playfair TJ, Mc Gill J, Jones BR, Steele AD Mc G. Amoeba infection of the eye. 1974; Lancet 2: 1537-40.

38. Jones DB. Polymicrobial keratitis. Trans Am Ophthalmol Soc. 1981; 79:153-67.

39. Cruz OA, Sabir SM, Capo H, Alfonso EC. Microbial keratitis in childhood. Ophthalmol 1993; 100: 192-6.

40. Hemady RK. Microbial keratitis in patients infected with the human immunodeficiency virus. Opthalmol 1995; 102: 1026-30.

41. Aristimuno B, Nirankari VS, Hemady RK, Rodrigues MM. Spontaneous ulcerative keratitis in immuncompromised patients. Am J Ophthalmol. 1993; 115: 202- 8.

42. Abbas KA, Lichtman AH, Pober JS. Cellular and molecular immunology. W B Saunders Company. 1997.

43. Sainz de la Maza M. Immunoregulation, Immune tolerance, autoimmunity, and immune ”privilege”. In Principals and practice of ophthalmology eds. Albert DM, Jacobiec FA, WB Saunders company 1994; 64 : 785-802.

44. Watt PJ. Pathogenetic mechanisms of organisms virulent to the eye. Trans Ophthalmol Soc UK. 1986;105: 26-31.

45. Wand M, Olive GM Jr, Mangiaracine AB. Corneal perforation and iris prolapse due to Mima polymopha. Arch Ophthalmol. 1975; 93: 239-241

46. Nassif X. Interaction between encapsulated Neisseria meningitidis and host cells Int Microbiol. 1999; 2: 133-6.

47. Lyczak JB, Cannon CL, Pier GB, Establischment of Pseudomonas aeruginosa infection: lesson from a versatile opportunist. Microb Infect. 2000; 2: 1051- 60.

48. Cheung J, Slomovic AR. Microbila etiology and predisposing factors among patients hospitalized for corneal ulceration. Can J Ophthalmol. 1995;30: 251-5.

49. Slusher MM, Myrvik QN, Lewis JC, Gristian HG. Extended-wear lenses, biofilm and bacterial adhesion. Arch Ophthalmol. 1987;105: 110-5.

50. Holden BA, Mertz GW, McNally JJ. Corneal swelling responses to contact lenses worn under extended wear conditions. Invest Ophthalmol Vis Sci 1983;24: 218-26.

51. Stehr-Green JK, Bailey TM, Visvesvara GS. The epidemic of Acanthamoeba keratitis in the United States. Am J Ophthamol. 1989; 107: 331-6.

52. O´Day D. Fungal keratits. in Ocular Infecction & Immunology. Eds. Peposo JS, Holland GN, Willhelmus KR. Moseby -Year Book Inc. 1996; 77:1048-61.

53. Alizadeh H, Niederkorn JY, Mc Culley JP. Acanthamoeba Keratitis. in Ocular Infecction & Immunology. Eds. Peposo JS, Holland GN, Willhelmus KR. Moseby -Year Book Inc. 1996; 78: 1062-71.

54. Huan AJW, Pflugfelder SC. Nocardial and Actinomyces keratitis. in Ocular Infecction & Immunology. Eds. Peposo JS, Holland GN, Willhelmus KR. Moseby -Year Book Inc. 1996; 76: 1043-47.

55. Wilhelmus KR, Liesegang TJ, Osato MS, Jones DB: Cumithech 13:A: Laboratory diagnosis of ocular infections. Washington, DC 1994. American Society of Ophthalmology

56. Mc Leod SD, Koahdouz-Isfahani A, Rostamina K, Flowers CW, Lee PP, McDonnell PJ. The role of smear, culture, and antibiotic sensitivity testing in the management of suspected infectious keratits. Ophthalmol. 1996; 23-8.

57. Kowal VO, Levey SB, Laibson PR, Rapuano CJ, Cohen EJ. Use of routine antibiotic sensitivity testing for the management of corneal ulcers. Arch Ophthalmol. 1997; 115: 462-5.

58. Mortlet N, Dart J. Routine antibiotic sensitivity testing for corneal ulcers. Arch Ophthalmol. 1998; 116: 1262.

59. Ficker L, Kirkness C, Mc Carney A, Seal D. Microbial keratitis-the false negative. Eye. 1991; 5: 549-59.

60. Mathers WD, Sutphim JE, Folberg R, Meier PA, Wenzel RP, Elgin RG. Outbreak of keratitis presumed to be caused by Acanthamoeba. Am J Ophthalmol. 1996;121: 129-42.

61. Lehman OJ, Green SM, Morlet N, Kilvington S, Keys MF, Matheson MM, Dart JK, McGill JI, Watt PJ. Polymerase chain reaction analysis of corneal epithelial and tear samples in the diagosis of Acanthamoeba keratitis. Invest Ophthalmol Vis Sci. 1998; 38: 1261- 5

62. Chen KH, Sheu MM, Lin SR. Rapid diagnosis of mycobacteria to the species level by polymerase chain reaction and restriction enzyme analysis- a case report of corneal ulcer. Kao Hsiung I Hsueh Ko Hsueh Tsa Chih. 1997; 13: 583- 8.

63. Illingworth CD, Cook SD. Acanthamoeba Keratitis. Surv Ophtalmol 1998; 42: 493-508.

64. O´Day DM, Head WS. Advances in the Management of Keratomycosis and Acanthamoeba Keratitis. Cornea. 2000; 19: 681-7.

65. O´Brien TP, Maguire MG,Fink NE. Efficacy of ofloxacin vs cefazolin and tobramycin in the therapy of bacterial keratitis. Arch Ophthalmol. 1995; 113:1257-65.

66. The ofloxacin study group. Ofloxacin monotherapy for the primary treatment of microbial keratitis: a double-masked, randomized, controlled trial with conventional dual therapy. Ophthalmology. 1997; 104: 1902-9.

67. Alfonso EC, Rosa RH. Fungal keratitis in: Cornea, eds:Krachmere JH, Mannis MJ, Holland EJ. Moseby-Year Book, Inc. 1997;2: 1253-65.

68. Hanada K, Shimazaki J, Shimmura S, Tsubota K. Multilayered amniotic membrane transplantation for severe ulceration of the cornea and sclera. Am J Ophthalmol. 2001; 131(3): 324-31.

69. Kirkness CM, Adams GG, Dilly PN, Lee JP. Botulinum Toxin A-induced protective ptosis in corneal disease. Ophthalmol. 1988; 95: 473-80.
