# Supplementary material for: Infectious bacterial keratitis management in a Swedish region: Risk factors, treatment and time‐to‐resolution
Source: Acta Ophthalmol. 2026 Apr 12;104(7):802–10. doi: 10.1111/aos.70141 (PMC13651938; doi:10.1111/aos.70141)
Supplement: Supplementary file 2 [file AOS-104-802-s002.docx]

## Supporting information 1, detailed patient selection process and microbiological sample collection.

**Figure S1, Patient selection process:**

Information was exported from the records in cases with one or more of the following ICD10 diagnosis codes: H16: Keratitis, H16.1: Other superficial keratitis without conjunctivitis, H16.2: Keratoconjunctivitis, H16.3: Interstitial and deep keratitis, H16.8: Other keratitis, H:16.9: Keratitis, unspecified, H19.2: Keratitis and keratoconjunctivitis in other infectious and parasitic diseases classified elsewhere, H19.3: Keratitis and keratoconjunctivitis in other diseases classified elsewhere. Figure 1 shows the process of selection of the data analysed in the current study. The initial search retrieved 2865 records with suspected infections keratitis. Bacterial keratitis was clinically diagnosed in 1270 patients, samples from 336 patients were sent to the clinical microbiology laboratory for culture confirmation. The laboratory analysis revealed 190 patients with cultured confirmed BK and 8 patients with *Candida* spp. The *Candida* spp. patients were excluded from further analysis as indicated in Figure 1, patients with combined fungal and bacterial infection were included. The culture positive rate was 59%.

**Microbiological sample collection:**

During direct inoculation, corneal samples were undertaken after the installation of topical unpreserved anaesthesia from the infiltrate with a sterile cotton swab and a 21-gauge sterile needle. The swab was then used to directly inoculate half of the area of two solid media (hematin and blood agar) and a 21-gauge sterile needle was used to inoculate the other half of the two solid media. For indirect inoculation, the same 21-gauge needle was submerged in fastidious anaerobic broth in a sterile fashion, which was later sub cultured onto agar plates for both anaerobic and aerobic incubation at the laboratory. Conjunctival samples were taken with the indirect inoculation method. Care was taken to avoid touching eyelids and a sterile cotton swab was used after the instillation of preservative-free topical anaesthetic drops. The cotton swab was brushed against the conjunctival surface. The tip of the cotton swab was submerged in transport media and sent to the laboratory.
