# Supplementary material for: Infectious bacterial keratitis management in a Swedish region: Risk factors, treatment and time‐to‐resolution
Source: Acta Ophthalmol. 2026 Apr 12;104(7):802–10. doi: 10.1111/aos.70141 (PMC13651938; doi:10.1111/aos.70141)
Supplement: Supplementary file 3 [file AOS-104-802-s003.docx]

## Supporting information 5, Cox regression without the 8 patients that received antibiotics prior to culture sampling.

**Table S3: Summary of the Cox proportional hazards regression model, with characteristics: age, sex, Gram-stain, aetiology, and antibiotic treatment - as independent predictors of TTR for BK in our sample with the 8 patient receiving antibiotics prior to culture excluded from the analysis.**

| **Characteristic** | **HR^1^** | **95% CI^2^** | **p-value** |
| --- | --- | --- | --- |
| **Age** | 0.99 | 0.98, 0.99 | **<0.001** |
| **Sex** |  |  |  |
| M (reference) | - | - |  |
| Female | 1.95 | 1.34, 2.85 | **<0.001** |
| **Gram-stain** |  |  |  |
| Gram-negative (reference) | - | - |  |
| Gram-positive | 2.44 | 1.44, 4.14 | **<0.001** |
| **Aetiology** |  |  |  |
| Miscellaneous/unknown (reference) | - | - |  |
| SI/B | 1.43 | 0.82, 2.48 | 0.2 |
| OSD | 1.43 | 0.81, 2.53 | 0.2 |
| CLW | 2.68 | 1.75, 4.09 | **<0.001** |
| **Antibiotic treatment** |  |  |  |
| No chloramphenicol treatment (reference) | - | - |  |
| Started with chloramphenicol | 0.95 | 0.61, 1.48 | 0.8 |
| Switched to chloramphenicol | 0.53 | 0.35, 0.81 | **0.003** |
