# Supplementary material for: Infectious bacterial keratitis management in a Swedish region: Risk factors, treatment and time‐to‐resolution
Source: Acta Ophthalmol. 2026 Apr 12;104(7):802–10. doi: 10.1111/aos.70141 (PMC13651938; doi:10.1111/aos.70141)
Supplement: Supplementary file 4 [file AOS-104-802-s004.docx]

## Supporting information 3, Cox regression including surgical intervention as predictor

**Table S1: Summary of the Cox proportional hazards regression model, with characteristics: age, sex, Gram-stain, aetiology, antibiotic treatment and surgical intervention - as independent predictors of TTR for BK in our sample.**

| **Characteristic** | **HR^1^** | **95% CI^2^** | **p-value** |
| --- | --- | --- | --- |
| **Age** | 0.99 | 0.98 0.99 | **0.006** |
| **Sex** |  |  |  |
| M (reference) | - | - |  |
| Female | 1.93 | 1.34, 2.79 | **<0.001** |
| **Gram-stain** |  |  |  |
| Gram-negative (reference) | - | - |  |
| Gram-positive | 2.47 | 1.48, 4.14 | **<0.001** |
| **Aetiology** |  |  |  |
| Miscellaneous/unknown (reference) | - | - |  |
| SI/B | 1.63 | 0.96, 2.78 | 0.070 |
| OSD | 1.38 | 0.79, 2.40 | 0.3 |
| CLW | 2.61 | 1.72, 3.96 | **<0.001** |
| **Antibiotic treatment** |  |  |  |
| No chloramphenicol treatment (reference) | - | - |  |
| Started with chloramphenicol | 1.07 | 0.69, 1.64 | 0.8 |
| Switched to chloramphenicol | 0.56 | 0.37, 0.84 | **0.006** |
| **Surgical intervention** |  |  |  |
| No (reference) | - | - |  |
| Yes | 0.50 | 0.33, 0.77 | **0.002** |

When surgical intervention was added as a predictor, surgical patients showed a slower healing rate than non-surgical patients, without affecting the outcome of the other predictors. However, this variable violated the proportional hazards assumption, so it was not included in the final analysis.
