# Supplementary material for: Infectious bacterial keratitis management in a Swedish region: Risk factors, treatment and time‐to‐resolution
Source: Acta Ophthalmol. 2026 Apr 12;104(7):802–10. doi: 10.1111/aos.70141 (PMC13651938; doi:10.1111/aos.70141)
Supplement: Supplementary file 5 [file AOS-104-802-s005.docx]

## Supporting information 4, Descriptive statistics of TTR and Cox proportional hazards regression model, excluding patients who underwent amniotic membrane transplant.

The median TTR of BK-infections, when excluding patients who underwent amniotic membrane transplant was 47 days (IQR: 16–101). Infections caused by Gram-negative species had a median TTR of 71 days (95%CI: 33, 209), compared to infections caused by Gram-positive species with a median TTR of 43 days (95%CI: 36, 62). Males had a median TTR of 69 days (95%CI: 41, 101), and females had a median TTR of 38 days (95%CI: 28, 54). Comparing the different aetiologies, contact lens wear (CLW) had a median TTR of 21 days; (95%CI: 12, 33). BK with miscellaneous or unknown (Misc./unknown) aetiologies had a TTR at 84 days (95%CI: 55, 143), and BK associated with ocular surface disease (OSD) that had a median TTR of 54 days (95%CI: 38, 232). Lastly BK in severely ill or blind (SI/B) patients had a TTR of 50 days (95%CI: 36, 141). Table S2 shows the Cox proportional hazards regression model for our sample excluding patients who underwent amniotic membrane transplant.

**Table S2: Summary of the Cox proportional hazards regression model, with characteristics: age, sex, Gram-stain, aetiology, and antibiotic treatment - as independent predictors of TTR for BK in our sample excluding patients who underwent amniotic membrane transplant.**

| **Characteristic** | **HR^1^** | **95% CI^2^** | **p-value** |
| --- | --- | --- | --- |
| **Age** | 0.99 | 0.98 1.00 | **0.002** |
| **Sex** |  |  |  |
| M (reference) | - | - |  |
| Female | 1.7 | 1.16, 2.48 | **0.006** |
| **Gram-stain** |  |  |  |
| Gram-negative (reference) | - | - |  |
| Gram-positive | 2.73 | 1.58, 4.70 | **<0.001** |
| **Aetiology** |  |  |  |
| Miscellaneous/unknown (reference) | - | - |  |
| SI/B | 1.55 | 0.87, 2.74 | 0.130 |
| OSD | 1.38 | 0.79, 2.41 | 0.3 |
| CLW | 2.94 | 1.91, 4.52 | **<0.001** |
| **Antibiotic treatment** |  |  |  |
| Started with chloramphenicol (reference) | - | - |  |
| Switched to chloramphenicol | 0.55 | 0.36, 0.84 | **0.006** |
| No chloramphenicol treatment | 0.89 | 0.57, 1.40 | 0.6 |
